# Anxiety and depression in Alzheimer’s disease: a systematic review of pathogenetic mechanisms and relation to cognitive decline

**DOI:** 10.1007/s10072-022-06068-x

**Published:** 2022-04-23

**Authors:** Rossana Botto, Nicoletta Callai, Aurora Cermelli, Lorenzo Causarano, Innocenzo Rainero

**Affiliations:** 1 Department of Neuroscience, University of Turin, Turin, Italy; 2 Clinical Psychology Unit, “Città Della Salute E Della Scienza Di Torino” Hospital of Turin, Turin, Italy; 3 Aging Brain and Memory Clinic, Department of Neuroscience, University of Turin, Turin, Italy; 4 Biblioteca Federata Di Medicina “Ferdinando Rossi”, University of Turin, Turin, Italy

**Keywords:** Anxiety, Depression, Alzheimer’s disease, Psychological symptoms, Dementia

## Abstract

**Objectives:**

To explore the pathogenetic hypothesis provided to explain the comorbidity of anxious and depressive symptomatology and AD and to assess the association between anxious and depressive symptoms and the AD-related cognitive impairment.

**Methods:**

In October 2020 and March 2021, PsycINFO, Embase, Ovid, and CINAHL were searched for peer-reviewed original articles investigating anxiety and/or depression in AD.

**Results:**

A total of 14,760 studies were identified and 34 papers on AD patients were included in the review. Suggested biological causes of depression and anxiety in AD include higher strychnine-sensitive glycine receptor (GlyRS) functioning and selective reduction of N-methyl-d-aspartate (NMDA) receptor NR2A density, cortical and limbic atrophy, lower resting cortical metabolism, lower CSF Aβ42 and higher t-tau and p-tau levels, and neuritic plaques. At the same time, dysthymia arises in the early stages of AD as an emotional reaction to the progressive cognitive decline and can cause it; anxiety can appear as an initial compensating behaviour; and depression might be related to AD awareness and loss of functional abilities. Affective symptoms and the expression of the depressive symptoms tend to reduce as AD progresses.

**Conclusion:**

The neurodegeneration of areas and circuits dealing with emotions can elicit anxiety and depression in AD. In the early stages of the disease, anxiety and depression could arise as a psychological reaction to AD and due to coping difficulties. In late AD stages, the cognitive impairment reduces the emotional responses and their expression. Anxiety and depression are more intense in early-onset AD, due to the major impact of AD on the individual.

**Supplementary Information:**

The online version contains supplementary material available at 10.1007/s10072-022-06068-x.

## Introduction

Alzheimer’s disease (AD) is a primary neurodegenerative dementia and one of the leading causes of disability in older people [1]. AD is clinically characterized by a progressive, global cognitive impairment that affects a person’s ability to perform everyday activities and is associated with brain changes that involve the extracellular accumulation of beta-amyloid plaques outside neurons and intraneuronal deposition of tau tangles inside neurons [2].

Although the core symptoms of AD are memory impairment and deficits in other cognitive domains [3], neuropsychiatric symptoms such as anxiety and depression are commonly observed during the clinical course of the illness [4, 5]. In AD, the prevalence of anxiety ranges from 9.4% (preclinical phase) to 39% (from mild to severe decline) [6, 7] and the prevalence of depression in mild-to-moderate AD varies from 14.8% [8] to 40% [9]. Anxiety is generally characterized by excessive worry, tenseness, irritability, wandering, and decreased engagement in once pleasurable activities [10]. Anxious symptoms seem to be associated with more severe impairments in activities of daily living and worse behavioural concerns [11], and anxiety could be considered a psychological response to AD diagnosis [12]. Typical depressive symptoms in AD are insomnia, social withdrawal, reduced purpose-oriented behaviour, loss of interest in once-enjoyable activities and hobbies, guilt, hopelessness, and sadness [13]. Anxiety and depression often overlap, especially in patients with mild AD [14].

Nevertheless, despite this evidence, due to progressive cognitive deterioration and to diagnostic and methodological difficulties in assessing anxiety and depression in AD patients, it is not easy to evaluate their role with respect to the progression of the disease.

Anxiety and depression in AD have been largely studied. Nevertheless, the pathogenetic explanations of the relationship of anxiety and depression with AD are still not so clear. On the one hand, a history of anxiety and depressive disorders, as well as their presence at the first stages of AD, represents a risk factor for the development of dementia [15, 16]. On the other hand, it is well-known that neuropsychiatric symptoms and AD share some common biological bases. Moreover, to our knowledge, to date, no studies proposed summary explanations of the various reasons why anxiety and depression can appear in comorbidity with AD and during its progression.

It is known that anxiety and depression can manifest during AD, and there are always causes explaining psychological symptoms. Thus, why AD patients can suffer from anxiety and depression? Which are the causes for the occurrence of these symptoms in patients with AD?

Research on the theme is heterogeneous relatively to the aims and the methodology of the studies. Most of the published studies on this theme had different aims but presented suggestions that answer to this clinical question in comment to their evidence. Moreover, singly consulted, they offer partial explanations to this clinical phenomenon that, instead, is complex. Furthermore, other reviews on anxiety and depression in AD did not specifically deal with the pathogenetic hypothesis on their comorbidity and suggest the analysis of the basic mechanisms explaining the prevalence of anxiety and depression in AD [6, 17, 18].

Therefore, according to these considerations, it could be useful to analyse deeper and resume literature evidence on this topic, combining different data and providing a bio-psycho-social frame to explain why AD patients can suffer from anxiety and depression. Hence, this systematic review had the aim to collect the evidence published to date that answer the above-presented clinical question. This question can be declined into two sub-hypotheses: anxiety and depression in AD could be due to pathogenetic mechanisms and there could be a relationship between them and cognitive decline. Thus, the purposes of this systematic review were to explore the pathogenetic hypothesis provided to explain the comorbidity of anxious and depressive symptomatology and AD and to assess the association between anxious and depressive symptoms and the AD-related cognitive impairment.

## Methods

The systematic review (PROSPERO registration n. CRD42019126592) was conducted according to the recommendations of the Preferred Reporting Items for Systematic Reviews and Meta-Analyses (PRISMA) criteria [19]. The study was approved by “Comitato Etico Interaziendale A.O.U. San Giovanni Battista di Torino A.O. C.T.O./Maria Adelaide di Torino”: protocol number 0034410, procedure number CS2/1179, date of approval: 29/03/19.

### Search strategy

The literature search was performed in October 2020 on PsycINFO, Embase, MEDLINE, and CINAHL databases. Then, a search update followed in March 2021 on the same databases. The search was conducted by L.C., a librarian expert in data extraction from databases, using keywords including the following terms (MeSH and free words): (“Alzheimer’s disease” [MeSH] OR (Alzheimer disease) OR (Alzheimer) OR Dementia [MeSH]) AND (“Anxiety” [MeSH] OR (Anxiety Disorder) OR (anx) OR “Depression” [MeSH] OR (Depressive Disorders) OR (Stress, Psychological)). See online resource “SM 1” for the details of the search strategy.

### Study eligibility criteria

Only full-text original articles published in English language on peer-reviewed journals were included in the search. In detail, studies with longitudinal, prospective, cross-sectional, multicentre, evaluative, or comparative designs, and not assessing interventions were analysed. Furthermore, studies had to be on patients with a diagnosis of AD. Diagnosis of AD had to be done using approved diagnostic criteria, i.e., those of DSM-III-R, DSM-IV, DSM-IV-TR, NIA-AA, NINCDS-ADRDA, ICD-10, CERAD, and Braak stage. No age-filters were used. Finally, they had to investigate symptoms of anxiety and/or depression; then, they had to propose pathogenetic hypothesis on anxiety and depression in AD and/or to investigate the association between anxiety and/or depression and cognitive impairment.

The exclusion criteria were (1) literature reviews; (2) systematic reviews; (3) meta-analysis; (4) case reports or case series studies; (5) clinical trials; (6) studies not including patients with AD (i.e., studies including patients with frontotemporal dementia, vascular dementia, Parkinson disease, mixed dementia, post-traumatic dementia, Huntington disease, dementia with Lewy bodies, and HIV-associated dementia); (7) studies not evaluating anxiety or depression; (8) studies evaluating anxiety or depression but not proposing pathogenetic hypothesis on anxiety and depression in AD or not investigating the association between anxiety and/or depression and cognitive impairment; (9) studies not published and not peer-reviewed; and (10) health policies and guidelines.

Studies of grey literature (theses, abstracts, books, dissertations) were not included in the search considering the high number of peer-reviewed articles published on the topic.

No limitations were established on publication data of the studies. No limitations were established on methods of anxiety and depression assessment on condition of these criteria: studies had to assess anxiety and/or depression using validated scales or structured interviews.

### Data collection and analysis

Two researchers (NC and AC) identified the potential studies of interest, screening titles, and abstracts. All the studies that did not meet all the inclusion criteria or that complied with almost one exclusion criterion were excluded.

Then, the remaining studies were screened reading their full text, and the selected articles were revised to verify whether they fulfilled the inclusion criteria. If there was any disagreement in the study selection, a third investigator (RB) read the article and NC, AC, and RB decided together for the inclusion of the article in the review or not.

All the studies selected for the review were revised considering the country, objectives, design, sampling and sample size, outcome measures, and results.

Any direct contact with authors was necessary with the following exception: for Panegyres et al.’s study [20], information was missing for the tools used to assess anxiety and depression. So, the people responsible for the C-Path Online Data Repository used by authors were contacted and they provided the missing information.

### Risk of bias

We assessed the quality of the included articles using the Newcastle–Ottawa Quality Assessment Scale (NOS) [21], a standardized instrument used to critically review nonrandomized studies with its design and content. Two independent investigators (A.C. and N.C.) analysed each article. Scores ranged from 0 to 9 points, with higher scores indicating higher study quality. We considered NOS scores of 0–3, 4–6, and ≥ 7 to indicate low, medium, and high quality, respectively.

## Results

The identified potentially relevant studies, resulting from the searches on the abovementioned databases, were 14,760. Of these, 4700 were on PsycINFO, 1351 on Embase, 4738 on Ovid, and 3971 on CINAHL. After comparison of the databases, 3888 duplicates were removed.

Screening titles and abstracts, 10,798 studies were identified as not meeting the inclusion criteria or fulfilling almost one exclusion criterion and were excluded.

Then, the full texts of the 74 articles considered relevant for the inclusion in the review were analysed. Of those, 40 were excluded because they did not meet the inclusion criteria, or they meet almost one exclusion criterion.

Finally, 34 papers were included in the systematic review after the end of the screening process.

The selection procedure is represented through a flow diagram in the online resource “SM 2.”

### Methodological aspects

#### Country

Fifteen studies were conducted in the USA [22–36], 2 in Germany [37, 38], 2 in the Netherlands [39, 40] (Banning et al. [35] conducted their study both in the USA and in Netherlands), 1 in Singapore [41], 2 in Japan [42, 43], 1 in Sweden [44], 2 in Argentina [45, 46], 3 in England [47–49], 1 in Australia [20], 2 in France [50, 51], 1 in Italy [52], 1 in China [53], and 1 in Norway [54].

#### Objectives

Of the 34 studies, 17 explored the pathogenetic hypothesis that explains the comorbidity of AD, anxiety, and/or depression. Among these, 4 evaluated the association between anxiety, depression, and AD biomarkers; 8 evaluated whether anxious and depressive symptomatology in AD arise after the diagnosis of AD or as a reaction to perceived cognitive decline. Ten studies investigated whether anxiety and/or depression can influence the cognitive functions, 3 evaluated the prevalence of depressive and anxiety symptoms, 2 assessed the trajectories of depression, 1 examined if depression is a risk factor for the onset of AD, and 1 study explored clinical differences between early-onset AD and late-onset AD patients.

#### Study design

Of the 34 studies, 12 were longitudinal studies, 9 were cross-sectional studies, 1 was a comparative study, 1 was a multicentric study, 5 were prospective studies, 2 were retrospective studies, 1 was a cohort observational study, 2 were prospective longitudinal studies, and 1 was a retrospective longitudinal study.

#### Sample

The samples consisted of 12,512 elderly patients with diagnosis of AD or with probable AD in total. All the screened studies included both genders, predominantly women (56.4%). Patients’ mean age was 73.82 ± 6.8 years (range = 57.7–86.2). Relevant variability among studies was observed in the number of participants. Sample sizes ranged from 20 to 3747 AD patients.

The AD duration varies between studies: in 14 studies, patients suffered from AD from less than 6 years, and in 5 studies, patients suffered from AD from at least 7 years. The authors of the remaining studies did not provide precise data on AD duration.

At the time of the assessment, most patients were in a mild-to-moderate AD stage. Only in 4 studies, participants were patients with severe AD [24, 39, 41, 47].

In addition to AD, patients also showed a comorbidity of depression and/or anxiety. Of all the samples, 18 consisted of subjects with AD and depression; 5 consisted of subjects with AD and anxiety; and 5 included subjects with a comorbidity of AD, and depressive and anxious symptoms. In the other samples, during the study, patients with AD developed depression or anxiety.

About the samples of the included studies, only 2 of them were from psychiatric settings [28, 38] and only 1 was from primary care [26]. Most samples were from dementia specialty clinics. Among them, 15 were from outpatient settings, 4 were inpatient settings, 2 nursing home settings, and 3 in-home settings. The other remaining studies, instead, did not provide further information on the sample.

#### Outcomes assessment

For AD diagnosis, 26 studies used criteria established by the NINCDS-ADRDA [55] and 1 study [36] also used NIA-AA criteria [56]. Two studies [38, 54], instead, used ICD-10 diagnostic criteria, while the Diagnostic and Statistical Manual of Mental Disorders third edition revised (DSM III-R) [57] and the fourth edition (DSM IV) [58] have been used in 6 studies, respectively, for AD diagnosis [29, 43, 44] and for dementia diagnosis [35, 36, 40]. In one study [39], AD diagnosis was neuropathologically confirmed in post-mortem using the Consortium to Establish a Registry for AD (CERAD) criteria [59].

Few studies [39, 41, 48] also performed the staging of the severity of AD neuropathology, using the Braak staging scheme [60]. For the assessment of the cognitive impairment, the Mini Mental State Examination (MMSE) [61] was used in almost all the studies.

Most studies evaluated anxiety and depression using the Neuropsychiatric Inventory (NPI) [62], the DSM III-R/DSM IV criteria [57, 58], the Hamilton Depression Rating Scale (HDRS) [63], and the Hamilton Anxiety Rating Scale (HARS) [64].

### Risk of bias assessment

The risk of bias assessment of the included studies showed high-quality design in most of our studies (25 out of 34), while the rest appeared with a medium risk of bias (9 out of 34). A closer analysis revealed that most of the research did not clarify the duration of the follow-up or how many patients concluded it; some articles lost “stars” within the “comparability category”; finally, several studies did not specify in detail the history of the investigated disease. The table in the online resource “SM 3” shows the results of the risk of bias assessment, specifying how many “stars” we evaluated in each included study.

### Results

#### Pathogenetic hypothesis for anxiety, depression, and AD comorbidity

Depression and anxiety occur throughout the AD course both due to brain damage and psychosocial factors [43, 49, 54].

Higher anxiety levels seemed to be associated with higher strychnine-sensitive glycine receptor (GlyRS) functioning and selective reduction of N-methyl-d-aspartate (NMDA) receptor NR2A density [41]. Anxiety in AD could be explained by the atrophy in the right precuneus and inferior parietal lobule and hyperperfusion of the bilateral anterior cingulate cortex [42]. Higher anxiety was associated with lower resting metabolism in the bilateral entorhinal cortex, anterior parahippocampal gyrus, left anterior superior temporal gyrus, and insula [30].

Depression was associated with AD pathology, i.e., lower CSF Aβ42 and higher t-tau and p-tau levels [36], to atrophy of the insula, the inferior frontal lobe, and the limbic neural networks, and to changes in the temporal and parietal regions, including supramarginal, superior and inferior temporal and fusiform gyri, right posterior cingulate and precuneus, locus coeruleus, and basal nucleus of Meynert [22, 35, 44, 47, 53]. Depression can be determined by decreased cortical metabolism, neuritic plaques, and neuronal damage in the temporal cortex that lead to disinhibition of the HPA-axis. On the other hand, the association between depression, HPA hyperactivity, and cardiovascular disease can determine AD progression [39]. AD development seems to stop the continuity of the depressive state due to impaired memory and executive control [32]. Affective symptoms and the expression of the depressive symptoms tend to reduce as AD progresses [48, 53]. Dysthymia was primarily in the early stages of AD, as an emotional reaction to the progressive cognitive decline, while major depression may be related to biological factors and is a symptom of the neurodegenerative AD processes [26, 38, 45].

Banning et al. [35] explained anxiety in AD as an initial compensating behaviour, while depression could be more related to AD awareness or to the psychological reaction to AD, and to relational and biological factors [36]. Depression can also be reactive to AD-related loss of functional ability [29, 31, 33].

**Fig. 1:**
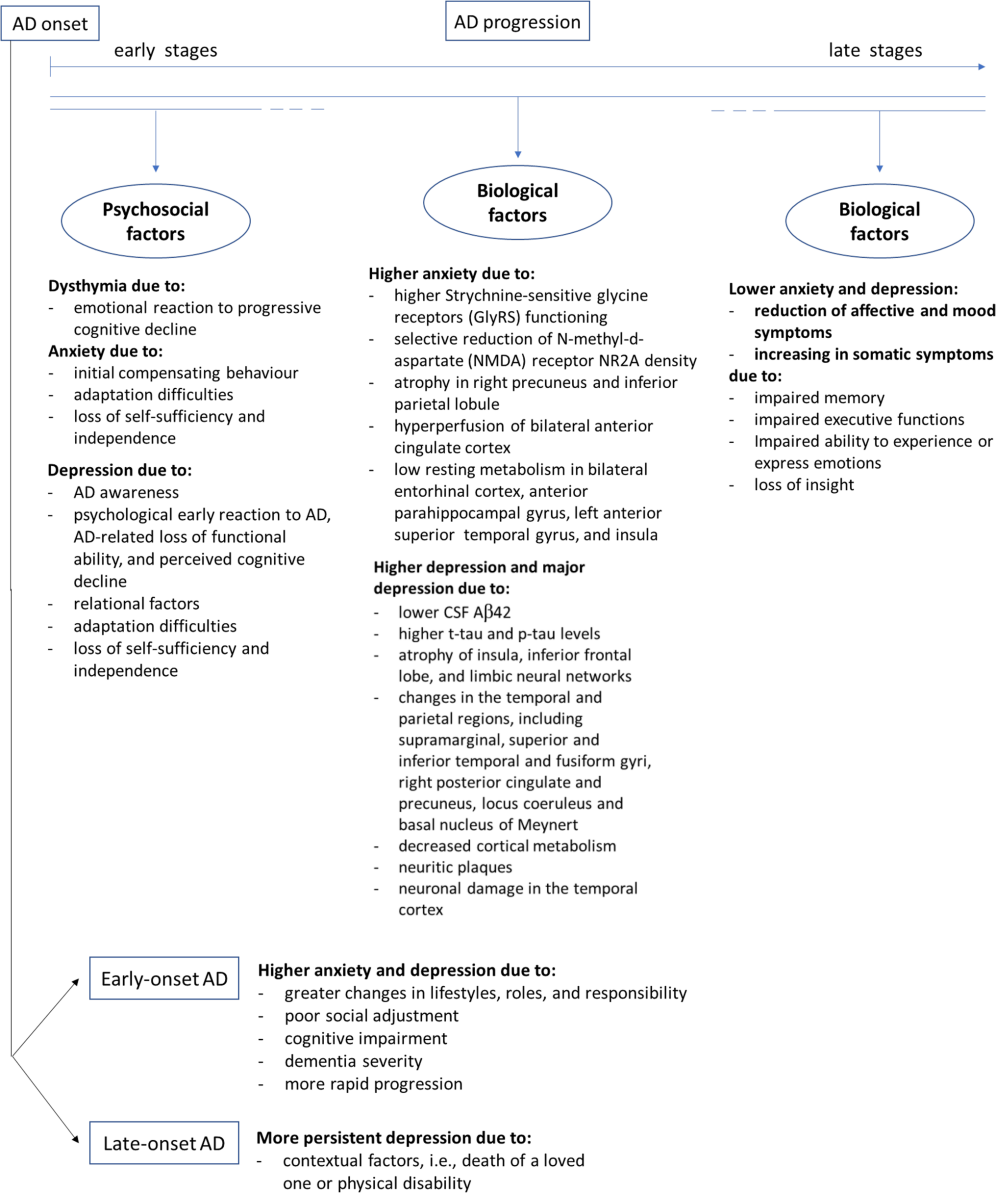
Causal factors for anxiety and depression in AD

Anxiety and depression were higher in patients with early-onset AD compared to patients with late-onset AD due to the following factors present in early-onset AD patients: greater changes in lifestyles, roles, and responsibility; poor social adjustment; cognitive impairment; dementia severity; and more rapid progression [20, 34, 43, 49]. Van Vliet [40] evidenced that in late-onset AD, depression was more persistent and was the most prevalent symptom, probably due to contextual factors, i.e., death of a loved one or physical disability (see Fig. 1).

#### Association between anxiety, depression, and cognitive impairment

Cognitive impairment seemed to mediate the association between the presence of anxiety, lower CSF levels of Ab42, and higher levels of CSF t-tau and p-tau [35]. Lower inhibition performance on Stroop test was associated with subsequent higher risk of anxiety and depression, due to the involving of anterior cingulate cortex [51]. Association of depressive symptoms and cognitive impairment seems to be independent of cortical plaques and tangles [25].

Depressive symptoms in AD were associated with a greater severity of cognitive impairment [26, 28, 37] and additional cognitive impairment, i.e., frontal planning impairments, disappear or improve with remission of depression [52].

Depression did not increase as mild cognitive impairment developed [32]. It could be an early reaction to perceived cognitive decline [50].

Depression can cause cognitive impairment [48, 54], but it seems to have no impact on cognitive functions during the early and advanced stages of AD [48].

**Fig. 2:**
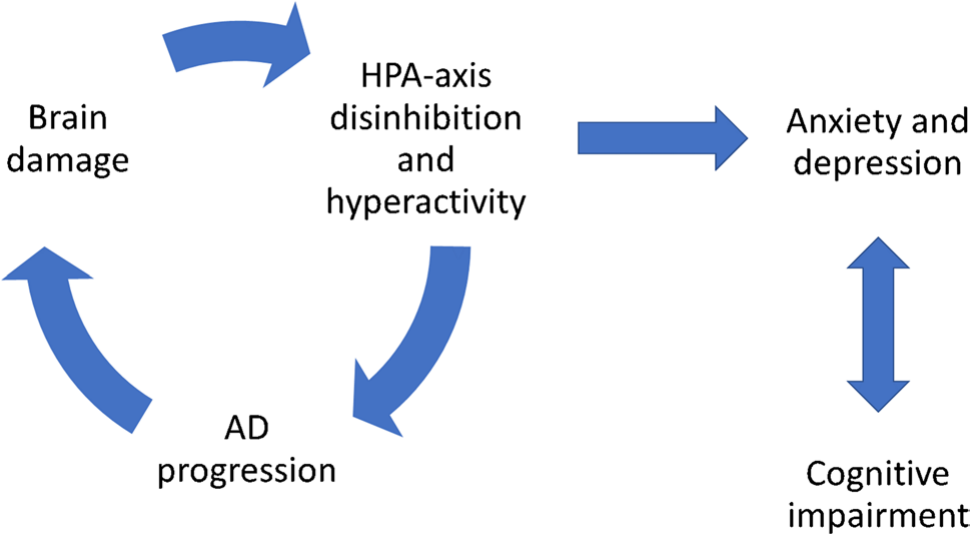
Relationships between anxiety, depression, AD, and cognitive impairment

Cognitive impairment was associated with a small reduction in mood symptoms and a modest increase in somatic symptoms: the AD progression determines the degradation of the ability to experience or express emotion [27] (see Fig. 2).

A summary of the 34 studies included in the systematic review is reported in Table 1.

**Table 1:** Characteristics of the included studies

Study		
Banning et al. [36] USA	Objective Study design Sample Outcomes Assessment Results	To assess depression over a 5-year follow-up period and to relate its trajectories to AD biomarkers Retrospective longitudinal study 3030 Pt with AD; gender: n.a.; age: nd.; dementia severity: prodromal AD criteria: DSM-IV-TR, NIA-AA, and NINCDS-ADRDA Cognitive ability assessment: MMSE Depression Assessment: NPI and NPI-Q Association between increasing probability of depression over time and AD pathology, i.e., lower CSF Aβ42 and higher t-tau and p-tau levels. Association between AD pathology and de novo or (initially) rising symptoms of depression
Banning et al. [35] USA	Objectives Study design Sample Outcomes Assessment Results	To study (inter)relations of AD biomarkers and neuropsychiatric symptoms in AD dementia and the impact of the cognitive functioning Retrospective cross-sectional study 626 Pt with AD; M: 299; F: 327; age: 73.9; dementia severity: mild level AD criteria: NINCDS-ADRDA and DSM-IV Cognitive abilities assessment: MMSE Anxiety and depression assessment: NPI, NPI-Q Association between the presence of anxiety and lower CSF levels of Ab42 and higher levels of CSF t-tau and p-tau, mediated by MMSE. No association between depression and Ab42 values, t-tau, p-tau, and reduced hippocampal volume (HCV) and between anxiety and HCV → Association of anxiety with AD pathology, due to impaired cognitive functioning, as initial compensating behaviour. Depression more related to psychosocial (i.e., awareness and psychological reaction to AD) or environmental (i.e., relationship with caregivers) or other biological factors (i.e., HPA axis or chronic inflammation)
Rouch et al. [51] France	Objective Study design Sample Outcomes Assessment Results	To evaluate the association between cognitive functioning and the occurrence of behavioural and psychological symptoms of dementia Prospective study. Pt were assessed at 6-, 12-, and 18-month follow-up 237 Pt with prodromal or mild AD; M: 100; F: 137; age: 79.5; age at onset: n.a.; dementia severity: mild level AD criteria: NINCDS-ADRDA Cognitive ability assessment: MMSE Anxiety and depression assessment: NPI Association between lower inhibition performance on Stroop test and subsequent higher risk of anxiety and depression → Stroop test involves anterior cingulate cortex that is involved in depression
Wu et al. [53] China	Objective Study design Sample Outcomes Assessment Results	To explore the probable neuroanatomical substrate of depressive symptoms of AD patients Cross-sectional study with control group 20 Pt with AD; M: 11; F: 9; age: 67.15; dementia severity: CDR 0.5–2 normal to moderate AD criteria: NINCDS-ADRDA Cognitive ability assessment: MMSE, ADL Anxiety and depression assessment: HAMA and HDRS Negative association between depression and insular and inferior frontal lobe grey matter volume, controlling for MMSE → Atrophy of the insula and the inferior frontal lobe more severe for depressed patients. Due to insular degeneration, depressive symptoms in AD could be related to abnormal somatic sensations; affective symptoms tend to reduce as AD progresses
Baillon et al. [49] England	Objective Study design Sample Outcomes Assessment Results	To compare neuropsychiatric symptoms (NPS) in people with early-onset Alzheimer’s disease (EOAD) and late-onset AD (LOAD) Retrospective 24 Pt with EOAD (< 65 years), M: 11; F: 13; 56 patients with LOAD (> 65 years), M: 20; F: 36; mean age: 59.3 EOAD, 82.3 LOAD. Duration of illness (months): 50.3 (41.8) EOAD, 26.9 (22.7) LOAD. Dementia severity: mild level AD criteria: NINCDS-ADRDA Cognitive ability assessment: MMSE, Bristol Activities of Daily Living Scale (BADLS) Anxiety and depression assessment: NPI Anxiety and depression higher in patients with EOAD compared to patients with LOAD → Diagnosis and AD-related changes in lifestyles, roles, and responsibility are greater in EOAD patients. Depression and anxiety occurring throughout the AD course both due to brain damage and psychosocial factors
Barca et al. [54] Norway	Objective Study design Sample Outcomes Assessment Results	To investigate the different trajectories of depressive symptoms among patients with AD and the relationship between the progression of AD and different trajectories Longitudinal observational study 282 Pt (177 with dementia due to AD and 105 with prodromal AD); M: 130; F:152; age: 73.2; AD duration: n.a.; dementia severity: mild level AD criteria: ICD-10; NINCDS-ADRDA Cognitive ability assessment: MMSE Depression assessment: Cornell Scale for Depression in Dementia and Clinical Dementia Rating Scale (CDR) Identified three distinct trajectories of depressive symptoms: stable low-average; high and decreasing; moderate and increasing. Association between the trajectories and AD progression: more depression in faster dementia → Faster progression leads to organic depression due to brain damages. Depression could also occur as a psychological reaction to greater impairments and can cause cognitive impairment
Tanaka et al. [43] Japan	Objective Study design Sample Outcomes Assessment Results	To investigate the relationship between dementia severity and behavioural and psychological symptoms in early-onset AD patients Cross-sectional 92 Pt with early-onset AD; M: 31; F: 61; age: 59.0; AD duration (years): 5.6; dementia severity: mild to severe AD criteria: NINCDS-ADRDA Cognitive ability assessment: MMSE Depression and anxiety assessment: Neuropsychiatric Inventory No differences in depression and anxiety among the three AD severity groups → Early-onset AD can lead to a poor social adjustment in society and family. Depression and anxiety are influenced by both psychosocial factors and advancing brain damage
Kaiser et al. [34] USA	Objective Study design Sample Outcomes Assessment Results	To evaluate anxiety in early-onset AD (EOAD) versus late-onset AD (LOAD) Cross-sectional 45 Pt (23 with EOAD, age: 57.68; 22 with LOAD, age: 80.32); M: 31; F: 14; AD duration (years): 3.09 (EOAD), 3.91 (LOAD); dementia severity: mild to moderate AD criteria: NINCDS-ADRDA Cognitive ability assessment: MMSE Anxiety assessment: NPI Higher anxiety in early-onset AD pts than in late-onset AD pts. Association between anxiety, gender, MMSE, and separation from caregivers in early-onset AD pts. Association between anxiety and psychotic and activating psychiatric symptoms related to dementia progression in late-onset AD pts → Men with early-onset AD are in midlife and have many roles and responsibilities. Thus, early-onset AD has higher impact on the individual than late-onset AD
Lebedeva et al. [44] Sweden	Objective Study design Sample Outcomes Assessment Results	To examine the association between depressive symptoms and neuroanatomical changes, brain structure and cerebrospinal fluid AD biomarkers Cohort observational study 189 AD Pt with and without depression; M: 94; F: 95; age: 71,1; AD duration (years): n.a.; dementia severity: n.a AD criteria: NINCDS-ADRDA and DSM-IV/ICD-10 Cognitive ability assessment: MMSE Depression assessment: Cornell Scale for Depression in Dementia and Geriatric Depression Scale Association between depression and changes in the temporal and parietal regions, including supramarginal, superior and inferior temporal and fusiform gyri, right posterior cingulate, and precuneus. Correlation between cortical thickness and t-τ was greater in depressed pts. in precuneus and parahippocampal cortex → More neurodegeneration on limbic structures in depressed AD patients. Disruptions of limbic neural networks can explain depression
Panegyres et al. [20] Australia	Objective Study design Sample Outcomes Assessment Results	Exploring clinical differences between early-onset AD patients and late-onset AD patients Cross-sectional study 3747 AD Pt with anxiety or depression (614 with early-onset AD; 3133 with late-onset AD); M: 1686; F: 2061; age: 59.3 (early-onset AD) 76.2 (late-onset AD); AD duration: n.a.; dementia severity: n.a AD criteria: McKhann criteria Cognitive ability assessment: n/a Anxiety and depression assessment: NPI, EQ-5D-3L, and GDS Early-onset AD pts were more likely to have anxiety or depression than late-onset pts. → They might be related to dementia severity and more rapid progression
Tagai et al. [42] Japan	Objective Study design Sample Outcomes Assessment Results	To investigate how anxiety in AD is related to the structure and function of the brain Retrospective study 26 Pt with probable AD; M: 6; F: 20; age: 74.95; AD duration: 27 months; dementia severity: mild level AD criteria: NINCDS-ADRDA; MRI; SPECT Cognitive function assessment: MMSE; CDR-SB; FAB Anxiety assessment: Behave-AD Association between anxiety, atrophy in the right precuneus (Pcs) and inferior parietal lobule (IPL), and hyperperfusion of the bilateral anterior cingulate cortex ACC → Pcs, IPL, and ACC belong to the fear neurocircuitry and are involved in anxiety disorders. In AD, their degeneration could explain anxiety
Zahodne et al. [33] USA	Objective Study design Sample Outcomes Assessment Results	To investigate longitudinal associations between functional abilities, cognitive status, and depressive symptoms in AD. Longitudinal study. Pt were assessed prospectively at 6-month intervals for up to 16 years, with an average of 5.5 years 517 Pt with probable AD; M: 222; F: 295; age: 74.19; AD duration: n.a.; dementia severity: mild level AD criteria: NINCDS-ADRDA Cognitive ability assessment: mMMSE; BDRS Depression assessment: CUSPAD Not worsening of depressive symptoms over AD course. Depressive symptoms predicted functional decline and vice versa → Depression may be reactive to the loss of functional ability, but it is not only a reaction to cognitive decline
Spalletta et al. [52] Italy	Objective Study design Sample Outcomes Assessment Results	To investigate cognitive progression of AD patients with or without major depressive episode Longitudinal study 119 newly diagnosed probable AD patients. M: 52; F: 67; age: 74.7; AD duration: 2.4 years; dementia severity: mild severity AD criteria: NINCDS-ADRDA Cognitive ability assessment: Mental Deterioration Battery, MMSE Depression assessment: a structured interview following modified DSM-IV diagnostic criteria for major depressive episode in AD More MMSE decline in pts with persistent depression, with incident depression over follow-up and in never depressed pts than in pts with recovered depression. → Depression in AD can be linked to additional cognitive impairment in AD, i.e., frontal planning impairments, that disappear or improve with remission of depression
van Vliet et al. [40] Holland	Objective Study design Sample Outcomes Assessment Results	To assess neuropsychiatric symptoms in young-onset AD (YO-AD)* and late-onset AD (LO-AD) Prospective cohort study Pt were assessed every 6 months for 2 years 221 Pt: 98 with YO-AD (age of onset before 65), 123 with LO-AD; M: 99; F: 122; age: 70; AD duration (years): 5.8 (YO-AD) 2.9 (LO-AD); dementia severity: moderate level AD criteria: 4th edition of the *Diagnostic and Statistical Manual of Mental Disorders, Text Revision* (2000) and the Dutch Consensus guidelines Cognitive ability assessment: MMSE Depression and anxiety assessment: Neuropsychiatric Inventory Depression and anxiety prevalence is lower in young-onset AD than in late-onset AD Depression is the most prevalent symptom in late-onset AD, and it decreases over time. Depression is less persistent in young-onset AD than in late-onset AD → Non-memory phenotype is associated with less atrophy of the medial temporal lobe, so with less symptoms. In oldest patients, contextual factors, i.e., death of a loved one or physical disability, and cerebrovascular diseases may increase depression
Meynen et al. [39] Netherlands	Objective Study design Sample Outcomes Assessment Results	To investigate the relationship between depressive state and neuropathological hallmarks of AD Prospective longitudinal study. Pt were evaluated every 6 months during the last years of their lives 43 Pt with possible or probable AD; M: 10; F: 33; age: 82.8; AD onset: 73.7 years; AD duration: 9.1 years; age of death: 82.8; dementia severity: severe level AD criteria: NINCDS-ADRDA; DSM-III-R; Braak stage for tangles Cognitive function assessment: MMSE at baseline; GDS and FAST at 6-month intervals Depression Assessment: CSDD Association between depression and the density of neuritic plaques in the entire cortex, and stronger in the temporal cortex, independently from clinical dementia and AD duration → Decreased cortical metabolism could determine both neuritic plaques and depression. The presence of neuritic plaques in the cortex might contribute, possibly through a toxicity of Aβ-amyloid, to the occurrence of depression. The neuronal damage in the temporal cortex could lead to disinhibition of the HPA-axis that contributes to depression. Vice versa, depression, associated to HPA hyperactivity and cardiovascular disease, may contribute to AD progress
Wilson et al. [32] USA	Objective Study design Sample Outcomes Assessment Results	To characterize change in depressive symptoms before and after the onset of dementia in AD Longitudinal study 1. Subgroup was evaluated every 3 years for a mean of 8 to 9 years 2. Subgroup: annual evaluation, for a mean of 3 years 1. Group: 357 Pt who developed AD during the study; M: 141; F: 216; age: 82.5 years; dementia severity: mild level 2. Group: 340 Pt (107 with AD, 81 with MCI, 152 with no cognitive impairment); M: 140; F: 200; age: 81.27 years AD criteria: NINCDS-ADRDA Cognitive function assessment: MMSE Depression assessment: (1) group: CES-D (self-report) v (2) group: HDRS (informant report) No change in depression during 2 to 3 years of observation after the diagnosis except for a slight decrease in positive affect → AD has little systematic effect on depression; since AD develops, impaired memory and executive control seem to stop the continuity of the depressive state
Amieva et al. [50] France	Objective Study design Sample Outcomes Assessment Results	To examine the emergence of the first clinical symptoms over a 14-year period of follow-up before the dementia phase of AD Longitudinal study. Subjects were evaluated at home at the initial visit and at 1, 3, 5, 8, 10, 13, and 15 years 350 AD Pt.; M: 243; F: 107; age: 86.2; AD duration: n.a.; dementia severity: n.a AD criteria: NINCDS-ADRDA Cognitive ability assessment: a battery of tests including MMSE Depression assessment: Center for Epidemiologic Studies-Depression scale (CESD) Association between global deficits and depressive symptoms. → Depression could be an early reaction to perceived cognitive decline
Wilson et al. [31] USA	Objective Study design Sample Outcomes Assessment Results	To test the hypothesis that depressive symptoms increase during the prodromal phase of AD Prospective cohort study 190 Pt with incident AD, M: 54; F: 136; age: 80.0; AD duration (years): 3.9; dementia severity: n.a AD criteria: NINCDS-ADRDA Cognitive ability assessment: MMSE Depression assessment: CES-D No significant changes in depressive symptoms after the AD diagnosis, although symptoms tended to decrease in women relative to men and in those with a higher premorbid level of openness and a lower premorbid level of agreeableness. Not increasing in depression as mild cognitive impairment developed → Depressive symptoms might influence the relation of the AD pathologic changes to cognition. Depression was associated with sex and personality, and some people experience a depressive reaction to AD
Tsang et al. [41] Singapore	Objective Study design Sample Outcomes Assessment Results	To correlate several glutamatergic measures with chronic anxiety in AD Prospective longitudinal study 21 Pt with AD (10 with low anxiety—LA; 11 with high anxiety—HA); M: 6; F:15; age: 77.4; age at onset: 70.45; age at death: 79.85; AD duration: 9.4 years Dementia severity: severe cognitive impairment AD criteria: CERAD scale Cognitive function assessment: MMSE; ADL Anxiety/depression assessment: NPI Higher binding affinity to glycine recognition sites (GlyRS) in pt with higher anxiety. Association between higher GlyRS affinity, selective reduction of N-methyl-d-aspartate NMDA receptor NR2A density, and elevated anxiety → The development of anxiety in AD can have a neurochemical basis. Changes in the NMDA receptor complex can lead to GlyRS hyperfunction that lead to anxiety
Hashimoto et al. [30] USA	Objective Study design Sample Outcomes Assessment Results	To investigate the association between anxiety and regional glucose metabolism in AD Observational, cross-sectional 41 Pt: M: 35, F: 6; age: 75.5 years; AD: 2.8 years AD criteria: NINCDS-ADRDA Cognitive ability assessment: MMSE Anxiety and depression assessment: NPI Higher anxiety associated with lower resting metabolism in the bilateral entorhinal cortex, anterior parahippocampal gyrus, and left anterior superior temporal gyrus and insula → Reduced functional activity in the bilateral anterior inferomedial temporal cortex may contribute to anxiety in AD, independently from cognitive decline
Cannon-Spoor et al. [28] USA	Objective Study design Sample Outcomes Assessment Results	To examine the effect of history of major depressive disorder (MDD) on cognitive performance in AD patients Multi-site prospective study 43 Pt with AD (22 AD + MDD; 21 AD − MDD); M: 16; F: 27. Age: 69; age at onset of AD: 66.5; AD duration: 3.35 years Dementia severity: mild-to-moderate cognitive impairment AD criteria: NINCDS-ADRDA; CDR Cognitive function assessment: test battery consisting of MMSE, WAIS-R, Mattis Initiation/Perseveration subscale, Buschke SRT, Fluency task Depression Assessment: CADD A history of depression in AD Pt was associated with more severe cognitive deficits → MDD and AD may share some underlying genetic diathesis or may share risk factors. The experience of MDD, particularly if long-lasting or repeated, may result in an insult that increases the risk for AD or increases the severity of cognitive symptoms
Holtzer et al. [29] USA	Objective Study design Sample Outcomes Assessment Results	To examine the temporal relationship between depressive symptoms, function, and cognitive status in Pt with probable AD Multicentre cohort study Pt were followed for up to 14 years and evaluated every 6 months 536 Pt with probable AD (210 with AD and depression) M: 220; F: 316; age: 74; AD duration: 4.06 years Dementia severity: mild level of cognitive and functional impairments AD criteria: NINCDS-ADRDA; DSM-III-R Cognitive and functional ability assessment: 3MS; BDRS Depression assessment: CUSPAD Depression was associated with functional impairment but not with cognitive impairment. Decline in function, indeed, preceded the first episode of depressive symptoms → Lower functional activity may be a risk factor for the onset of depressive symptoms in AD
Milwain et al. [48] UK	Objective Study design Sample Outcomes Assessment Results	To investigate whether depression may influence the clinical expression of AD, analysing the relationship between cognition, the neuropathological stages of AD, and depressive symptoms Longitudinal study Pt with cognitive impairment were evaluated every 6 months, annually for those without 89 Pt with AD (48 with depression) M: 39; F: 50; age: 79.4 Dementia severity: 14.24% Pt in the pre-clinical stage of AD, 21.36% in the intermediate stages of AD, 43.61% in the final stages of AD AD criteria: Braak stage Cognitive function assessment: CAMCOG Depression assessment: CAMDEX Depression was associated with a more severe cognitive impairment, but only in intermediate stages of AD. Indeed, depressive symptoms had no impact during the early and advanced stages of AD → Depressive symptoms may contribute to the cognitive decline of AD Pt. The low prevalence of depression in the final stages of AD may be due to the fact that demented Pt are not able to express the depressive disorder
Gilley et al. [27] USA	Objective Study design Sample Outcomes Assessment Results	To evaluate factors related to the development of depressive symptoms in Pt with AD Longitudinal study. Pt were evaluated at baseline and at up to 4 annual follow-up examinations 410 Pt with AD, M: 136; F: 274; age: 75.5; age duration: n.a Dementia severity: moderate level at the study onset and a severe level at the last evaluation AD criteria: McKhann criteria Cognitive function assessment: MMSE Depression assessment: HRS-D + quantitative structured interviews with family members Premorbid neuroticism personality was associated with an increased rate of depressive symptoms in AD Pt Cognitive impairment was associated with a small reduction in mood symptoms and a modest increase in somatic symptoms → Decline in mood symptoms with increasing severity of cognitive impairment in Pt with AD can be explained as the result of the degradation of the ability to experience or express emotion as severity of AD increases
Wilson et al. [25] USA	Objective Study design Sample Outcomes Assessment Results	To study the relationship between depressive symptoms, clinical AD, and cognitive impairment Longitudinal study 130 elder participants, of whom 51 with probable AD AD criteria: NINCDS-ADRDA Cognitive ability assessment: 19 cognitive function tests Depression assessment: Center for Epidemiologic Studies Depression Scale (CES-D) Association of depressive symptoms with clinical AD and cognitive impairment seems to be independent of cortical plaques and tangles. → Depression-related glucocorticoid effects on neuronal function can contribute to risk of dementia. Depressive symptoms can also contribute to cognitive decline and clinical AD through some psychological mechanism
Zubenko et al. [26] USA	Objective Study design Sample Outcomes Assessment Results	To describe the prevalence and clinical features of the major depressive syndrome of AD, comparing Pt with AD to elderly Pt without AD Comparative study 243 Pt with probable AD. M: 100; F: 143. Age: 78.4; age at onset of AD: 69.0; dementia severity: moderate level 151 No-AD Pt. M: 70; F: 81; age: 70.9 AD criteria: NINCDS-ADRDA Cognitive function assessment: MMSE; CDR Depression assessment: HAM-D; CADD Higher lifetime prevalence of major depression among AD Pt and higher prevalence of MD in AD Pt with severe cognitive impairment → Premorbid major depressive episodes might increase the risk of developing AD, while prevalence of depressive episodes that occurred in the context of AD may be related to neurodegenerative events that contribute to the aetiology of major depression among AD Pt
Heun et al. [38] Germany	Objective Study design Sample Outcomes Assessment Results	To clarify the relationship between the age at onset of depression in relation to the onset of dementia Longitudinal study 57 Pt with AD and MD. M: 18; F: 39. Age: 74.2; age at onset of AD: 71.7; AD duration: n.a AD criteria: ICD-10 Cognitive function assessment: MMSE, SIDAM Depression assessment: CIDI Partial correlation between the onset of cognitive impairment and the onset of depression → Depression in AD might not be a symptom of psychological distress, but a symptom of the neurodegenerative process of AD that causes cognitive dysfunction as well
Chemerinski et al. [46] Argentina	Objective Study design Sample Outcomes Assessment Results	To examine the prevalence and correlates of generalized anxiety disorder (GAD) in AD Cross-sectional study 54 Pt with probable AD (18 with GAD compared with 36 Pt without anxiety disorder). M: 45%; F: 55%; age: 73.9; AD duration: 3.15 Dementia severity: mild (AD GAD ꞊ 39%; AD control ꞊ 72%), moderate (AD GAD ꞊ 44%; AD control ꞊ 22%), severe (AD GAD ꞊ 17%; AD control ꞊ 6%) AD criteria: NINCDS-ADRDA Cognitive function assessment: MMSE; CDR Anxiety assessment: DSM-III-R; SCID; SCID-II; HAM-D; HAM-A; Apathy Scale; Bech Mania Scale; PLACS GAD in AD was not associated with more severe cognitive impairment → GAD in AD may indicate a subsyndromal depressive state
Haupt, et al. [37] Germany	Objective Study design Sample Outcomes Assessment Results	To study the association between depression and severity of cognitive impairment in AD Longitudinal study. Pt were followed over 2 years with annual evaluations 78 Pt with AD. M: 21; F: 57. Age: 74.3; AD duration: 4.9 years; age at onset: 69.4; dementia severity: mild-to-moderate level AD criteria: NINCDS-ADRA; ICD-10 Cognitive function assessment: CAMDEX; CAMCOG; MMSE; GDS; DS; DBS Depression assessment: DMAS Depressive symptoms in AD Pt are in part associated with a greater severity of cognitive impairment → However, depressive symptoms are not prognostically relevant with respect to a lower or higher rate of symptom progression
Migliorelli et al. [45] Argentina	Objective Study design Sample Outcomes Assessment Results	To examine the prevalence, risk factors, and correlates of depression among patients with AD Cross-sectional study 103 Pt with probable AD, divided into three groups: major depression (*N* = 24), dysthymia (*N* = 29), and no depression (*N* = 50) M: 27; F: 76. Age: 73.2; AD duration: 4.3 years; age at onset: 68.9; dementia severity: mild (44.29%), moderate (47.38%), severe (14.42%) AD criteria: NINCDS-ADRDA Cognitive function assessment: MMS; WAB; TMT; WCST; BNT; TT; Digit Span Depression assessment: DSM-III-R; SCID; SCID-II; PSE; FH-RDC; HAM-D; HAM-A; FIM; STC High frequency of depression among Pt with probable AD. Specifically, dysthymia was primarily in the early stages of AD, and major depression was distributed across the different stages of the illness. No significant between-group differences in the severity of cognitive deficits → Dysthymia may be an emotional reaction to the progressive cognitive decline, while major depression may be related to biological factors
Förstl et al. [47] UK	Objective Study design Sample Outcomes Assessment Results	To examine the changes in the locus coeruleus (LC), substantia nigra (SN), basal nucleus of Meynert of AD Pt with and without depression and relate this to clinical features Prospective study 52 Pt with AD (14 with depression compared with 38 without depression) M: 12; F: 40; age: 83.2 AD duration: 8.2 (AD + D group) 7.5 (AD group without depression); dementia severity: severe cognitive impairment AD criteria: NINCDS-ADRDA Cognitive and functional ability assessment: MMSE, CAMCOG Depression assessment: GMSS, CAMDEX In AD Pt with depression, a lower neuronal count in the locus coeruleus and a higher in the basal nucleus of Meynert than AD Pt without depression were observed. There were no differences of the neuron numbers in the SN Pt with depression showed less cognitive impairment and their verbal skills were better preserved → The observed disproportionate loss of noradrenergic and cholinergic neurons in the LC and basal nucleus of Meynert may represent an important organic substrate of depression in AD
Lopez et al. [23] USA	Objective Study design Sample Outcomes Assessment Results	To evaluate the cognitive functions of patients (Pt) with probable AD and major depression in comparison with Pt with AD and no depression Longitudinal study. Two evaluations: at baseline and 1-year follow-up 10 Pt with AD and depression (developed after the onset of symptoms of dementia) compared with 10 nondepressed Pt with AD; M: 4; F: 16. Age (years): 67.45; AD duration (years): 3.2; dementia severity: mild level AD criteria: NINCDS-ADRA Cognitive function assessment: MMSE Depression assessment: DSM-III-R; HAM-D No association between depression and cognitive impairment. Pt with AD and depression did not manifest more severe neuropsychological impairments than Pt with AD without depression → Depression does not modify the neuropsychological features and the rate of progression of AD
Pearlson et al. [24] USA	Objective Study design Sample Outcomes Assessment Results	To study family history of affective disorder in AD Pt with first-episode depression Cross-sectional study 112 AD Pt (41 with depression) M: 34; F: 78; age: 68.9; AD duration: n.a.; dementia severity: severe cognitive impairment AD criteria: NINCDS-ADRDA Cognitive function assessment: MMSE Depression assessment: DSM-III; FH-RDC (in first and second-degree relatives) → The depressed patients had significantly more first- and second-degree relatives with depression than did control subjects Alzheimer’s disease as it evolves may interact with an existing genetic vulnerability to affective disorder, which is not expressed until the degenerative changes of Alzheimer’s disease unfold
Zweig et al. [22] USA	Objective Study design Sample Outcomes Assessment Results	To evaluate the pathological involvement of the locus coeruleus (LC), the dorsal raphe nucleus (DR), and the central superior (raphe) nucleus (CSN) in a series of aged control subjects and AD patients with or without depression Longitudinal study 22 AD Pt (8 with depression), AD duration: 7.75; + 12 aged control subjects; Age: 70.7; M: 16; F: 18 Dementia severity: moderate-to-severe cognitive impairment AD criteria: post-mortem histological evaluation Cognitive function assessment: MMSE Depression assessment: DSM-III Compared with control subjects, AD Pt showed higher levels of neuronal loss and higher counts of NFTs, particularly within the LC. Patients with AD complicated by major depression had fewer neurons at the mid-level of the LC and at the rostral level of the CSN in comparison with nondepressed patients → These findings demonstrate histological changes in the brain related to the presence of depression

Notes: *Young-onset AD is synonymous with early-onset AD and they both indicate AD onset before age 65. *3MS*, Modified version of the Mini-Mental State Examination; *ACC*, anterior cingulate cortex; *ACE-R*, Addenbrooke’s Cognitive Examination Revised; *ADL*, activities of daily living; *BDRS*, Blessed Dementia Rating Scale; *BPSD*, Behavioral and Psychological Symptoms of Dementia; *BEHAVE-AD*, Behavioural Abnormalities in AD Rating scale; *BMS*, Bech Mania Scale; *BNT*, Boston Naming Test; *Buschke SRT*, Buschke Selective Reminding Task; *CADD*, Clinical Assessment of Depression in Dementia; *CAMCOG*, Cambridge Cognition Examination; *CAMDEX*, Cambridge Examination for Mental Disorders of the Elderly; *CIDI*, Composite International Diagnostic Interview; *CDR*, Clinical Dementia Rating; *CDR-SB*, Clinical Dementia Rating scale sum of boxes; *CERAD*, Consortium to Establish a Registry for AD; *CES-D*, Center for Epidemiologic Studies Depression Scale; *CSDD*, Cornell Scale for Depression in Dementia; *CSF*, cerebrospinal fluid; *CUSPAD*, Columbia University Scale for Psychopathology in Alzheimer’s Disease; *DBS*, Dementia Behaviour Scale; *DMAS*, Dementia Mood Assessment Scale; *DS*, Blessed Dementia Scale; *EOAD*, early-onset Alzheimer’s disease; *FAB*, Frontal Assessment Battery; *FAS*, Verbal Fluency; *FAST*, Functional Assessment Staging scale; *FH-RDC*, Family History Research Diagnostic Criteria; *FIM*, Functional Independence Measure; *GAD*, general anxiety disorder; *GDS*, Geriatric Depression Scale; *GDS*, Global Deterioration Scale; *GlyRS*, Glycyl-tRNA synthetase; *HADS-A*, Hospital Anxiety and Depression Scale-Anxiety subscale; *HAM-A*, Hamilton Anxiety Rating Scale; *HAM-D*, Hamilton Depression Rating Scale; *HDRS*, Hamilton Depression Rating Scale; *HRS-D*, Hamilton Rating Scale for Depression; *iADL*, instrumental activity of daily living; *ICD-10*, International Classification of Diseases Tenth Revision; *IPL*, inferior parietal lobule; *MD*, major depression; *MDRS*, Mattis Dementia Rating Scale; *LOAD*, late-onset Alzheimer’s disease; *MMSE*, Mini Mental State Examination; *mMMSE*, Modified Mini-Mental State Exam; *MRI*, magnetic resonance imaging; *NIAA*, National Institute on Aging-Alzheimer’s Association criteria; *NINCDS-ADRDA*, National Institute of Neurological and Communicative Disorders and Stroke and the Alzheimer’s Disease and Related Disorders Association criteria; *NMDA*, N-methyl-d-aspartate; *NPI*, Neuropsychiatric Inventory; *NPI-Q*, Neuropsychiatric Inventory Questionnaire; *Pcs*, precuneus; *PDC-Dad*, Provisional Diagnostic Criteria for Depression of Alzheimer’s disease; *PLACS*, Pathological Laughing and Crying Scale; *PSE*, Present State Examination; *SCID*, Structured Clinical Interview for DSM; *SCID-II*, Structured Clinical Interview for DSM-III-R-Personality Disorders; *SPECT*, single-photon emission computed tomography; *SIDAM*, Structured Interview for the Diagnosis of Dementia of the Alzheimer’s type, multi-infarct dementia, and dementias of other etiologies; *SRT*, Buschke selective reminding test; *STC*, Social Ties Checklist; *TMT*, Trail Making Test; *TT*, Token Test; *WAB*, Western Aphasia Battery; *WAIS-R*, Wechsler Adult Intelligence Scale; *WCST*, Wisconsin Card Sorting Test

## Discussion

The review aims to provide a bio-psycho-social frame to explain why AD patients can suffer from anxiety and depression, considering the phenomenon in its complexity. Thus, multiple pieces of punctual evidence have been combined to resume and conceptualize the literature state of the art on reasons underlying the comorbidity between anxiety, depression, and AD. For this reason, the results of the studies analysed in this review are heterogeneous because they consider not only biological, but also psychosocial aspects and they deal with AD in all its different phases relative to its progression. They evidence that both anxiety and depression could be due to brain damage on the one hand and psychosocial factors on the other.

The neurodegeneration of neural areas and circuits dealing with emotions can determine hyperactivation and disinhibition of the HPA axis that can elicit the anxious and depressive symptoms. In this regard, it has been hypothesized that hyper perfusion and atrophy of cerebral areas—i.e., anterior cingulate cortex, precuneus and parietal lobule—but also receptor alterations—i.e., NMDA receptor—could be linked to depressive symptoms [41, 42]. This condition of chronic distress, in turn, can lead to neurodegeneration and contribute to AD progression. Then, during the late AD stages, the high rate of brain damage could stop the depressive condition due to the impairment of memory and the executive functions, and the intensity and expression of the affective symptoms are reduced. This phenomenon could be present because a more severe cognitive decline is frequently associated with loss of insight in AD [65]. Stronger negative emotions require a good cerebral function: if the cognitive impairment is too serious, patients will not be able to produce this type of emotional response [27, 37].

On the other hand, the early stages of AD seem to be characterized by dysthymia, as an emotional reaction to the cognitive decline, and anxiety, as an initial compensating behaviour. During the initial phases of AD, depressive symptoms can manifest due to AD awareness, the impairment of the socio-relational functioning, and the loss of functional abilities. Thus, in this phase, anxiety and depression could arise due to difficulties in the adaptation to the disease and represent the psychological response to the loss of self-sufficiency and independence [14, 15, 35]. Instead, major depression could be more related to biological factors, such as neurotransmitters and endocrine alterations or cortical apoptosis.

Literature evidence also differences in anxiety and depression between early-onset and late-onset AD. They seem to be more intense in early-onset AD due to more changes in lifestyles and life roles, to more responsibilities to cope with and to poorer social adjustment to the disease. Moreover, early-onset AD is often characterized by more rapid progression, dementia severity, and cognitive impairment and this can promote the psychological symptoms. The late-onset AD can occur in the elders in comorbidity with other concerns, such as physical disability or bereavements. Thus, in late-onset AD, depressive symptoms are the most prevalent and seem to be more persistent.

The results of the studies show an association between anxiety, depression, and cognitive decline. Anxious and depressive symptoms can manifest as an initial psychological reaction to the cognitive impairment. Furthermore, on its turn, depression can impact on the cognitive functioning, causing an increase of the cognitive deficits: i.e., during the remission of the depressive symptoms, the capacities of planning mediated by the frontal circuits improve. This role of influence of depression on cognition has been observed only in the intermediate AD phases.

Finally, higher cognitive impairment is associated with higher somatic symptoms of anxiety and depression and lower affective symptoms. This could be due to reduced metacognitive possibilities to experience and express emotions.

The methodology of the symptoms’ assessment could have moderated the abovementioned findings.

Assessment tools more specifically dedicated to the analysis of anxiety and depression in AD can estimate symptoms differently than those provided by more generic criteria, such as the DSM or the ICD [66, 67]. In this regard, Vilalta-Franch and colleagues [67] hypothesized that the degree of rigidity of the included criteria could influence the diagnosis. Furthermore, they observed that the use of the Neuropsychiatric Inventory (NPI) can be biased by the fact that caregivers usually overestimate patients’ depressive symptoms due to their own distress [68, 69].

The minority of studies performed longitudinal research designs, so further research with longitudinal observations is needed to better explore the variation of anxiety and depression along the AD course. Moreover, few studies assessed the AD severity, despite it emerging as a factor playing a role with respect to the considered psychological symptomatology. Another limitation of the studies is that they have used different AD diagnostic criteria, such as NINCDS-ADRDA, NIA-AA, DSM, and ICD. Furthermore, NINCDS-ADRDA, although it is the most used and more valid than the other diagnostic criteria, is less accurate than the currently used NIA-AA criteria. Large criteria for source search have been preferred to more stringent ones to collect and mix more evidence. At the same time, they reduced quality and homogeneity of evidence. In fact, NOS scores indicated that the quality of the included studies is not high, and studies were heterogeneous in aims, types of assessment, sampling, participants, AD duration, and neuropsychological assessment, that in most cases were not adequate. In this regard, also time impacted on method heterogeneity between older studies and the more recent ones, because the assessment criteria have changed and improved over time. Implementing more circumscribed reviews could preserve research quality and favour a more specific, detailed, and reliable comprehension of the described clinical phenomena. Moreover, most studies assessed anxiety and depression when AD was mild or moderate and not severe: this is understandable considering the serious cognitive decline that characterizes the AD advanced phases and hinders the psychological assessment, but, at the same time, it limits the exploration of anxiety and depression in patients with severe AD. Finally, AD is often present in comorbidity with dementias due to other causes, such as the vascular one. So, it would be useful to study anxiety and depression also in clinical condition of multiple dementias.

## Conclusion

The neurodegeneration of areas and circuits dealing with emotions can elicit the anxious and depressive symptoms that, in their turn, can lead to neurodegeneration. In the early AD stages, anxiety and depression could arise as a psychological reaction to the disease and due to difficulties in the adaptation to AD. During the late AD stages, the serious cognitive impairment reduces the emotional responses and their expression. Finally, anxiety and depression are more intense in early-onset AD, due to the major impact of AD on the individual.

Further research, especially with longitudinal design, considering all the AD stages, performing adequate neuropsychological assessment, and investigating psycho-social factors are needed to better clarify the comorbidity between depression, anxiety, and AD.

## Supplementary Information

Below is the link to the electronic supplementary material.

Supplementary file1 (DOCX 16 KB)

Supplementary file2 (DOCX 43 KB)

Supplementary file3 (DOCX 42 KB)

## Data Availability

Data sharing is not applicable to this article as no datasets were generated or analysed during the current study.
