# Supplementary material for: Anxiety and depression in Alzheimer’s disease: a systematic review of pathogenetic mechanisms and relation to cognitive decline
Source: Neurol Sci. 2022 Apr 23;43(7):4107–24. doi: 10.1007/s10072-022-06068-x (PMC9213384; doi:10.1007/s10072-022-06068-x)
Supplement: Supplementary file 1 — Supplementary file1 (DOCX 16 KB) [file 10072_2022_6068_MOESM1_ESM.docx]

**Title:** Anxiety and depression in Alzheimer’s disease: a systematic review of pathogenetic mechanisms and relation to cognitive decline.

**Journal name:** Neurological Sciences

**Authors and affiliations:**

**Rossana Botto^1,2^, Nicoletta Callai^2^, Aurora Cermelli^3^, Lorenzo Causarano^4^, Innocenzo Rainero^3^**

^1^Department of Neuroscience, University of Turin, Torino, Italy

^2^Clinical Psychology Unit, “Città della Salute e della Scienza di Torino” Hospital of Turin, Torino, Italy

^3^Aging Brain and Memory Clinic, Department of Neuroscience, University of Turin, Torino, Italy

^4^Biblioteca Federata di Medicina “Ferdinando Rossi”, University of Turin, Torino, Italy

**Corresponding author’s e-mail:**

rossana.botto@unito.it

**SEARCH STRATEGY**

**OVID**

1 exp *Alzheimer Disease/ (77523)

2 alzheimer.mp. (108197)

3 exp *Depression/ (75930)

4 exp *Anxiety/ or exp *Anxiety Disorders/ (100917)

5 anx*.mp. (266002)

6 exp *Depressive Disorder/ (86648)

7 (1 or 2) and (3 or 4 or 5 or 6) (3092)

8 exp *Stress, Psychological/ (87215)

9 (1 or 2) and (3 or 4 or 5 or 6 or 8) (3444)

10 limit 9 to (english and humans and observational study) (32)

11 limit 9 to (english and (adaptive clinical trial or clinical trial, all or clinical trial or comparative study or controlled clinical trial or evaluation studies or journal article or meta analysis or multicenter study or observational study or pragmatic clinical trial or randomized controlled trial or "review" or "systematic review")) [Limit not valid in Ovid MEDLINE(R),Ovid MEDLINE(R) Daily Update,Ovid MEDLINE(R) In-Process,Ovid MEDLINE(R) Publisher; records were retained] (3024)

12 "9706453".fc_acno. (1)

13 exp *Dementia/ (141625)

14 (1 or 2 or 13) and (3 or 4 or 5 or 6 or 8) (6905)

15 limit 14 to (english and (adaptive clinical trial or clinical trial, all or clinical trial or comparative study or controlled clinical trial or evaluation studies or journal article or meta analysis or multicenter study or observational study or pragmatic clinical trial or randomized controlled trial or "review" or "systematic review")) [Limit not valid in Ovid MEDLINE(R),Ovid MEDLINE(R) Daily Update,Ovid MEDLINE(R) In-Process,Ovid MEDLINE(R) Publisher; records were retained] (5907)

16 limit 15 to yr="2019 -Current" (596)

**EMBASE**

('alzheimer disease'/exp/mj OR alzheimer OR 'dementia'/exp/mj) AND ('major depression'/exp/mj OR 'anxiety disorder'/exp/mj OR 'anxiety'/exp/mj OR anx* OR 'depression'/exp/mj OR 'mental stress'/exp/mj) AND ([cochrane review]/lim OR [systematic review]/lim OR [meta analysis]/lim OR [controlled clinical trial]/lim OR [randomized controlled trial]/lim) AND [english]/lim AND ([embase]/lim OR [pubmed-not-medline]/lim) AND [2019-2021]/py

**PSYCINFO**

S13 S9 AND S10 Limiters - Publication Year: 2019-2021 View Results (574)

S12 S9 AND S10 Narrow by Language: - english View Results (6,356)

S11 S9 AND S10 View Results (6,747)

S10 S4 OR S5 OR S6 OR S7 OR S8 View Results (406,950)

S9 S1 OR S2 OR S3 View Results (91,601)

S8 MM "Psychological Stress" View Results (7,158)

S7 depressive disorder View Results (78,829)

S6 anx* View Results (286,094)

S5 MM "Anxiety" OR MM "Anxiety Sensitivity" OR MM "Computer Anxiety" OR MM "Death Anxiety" OR MM "Health Anxiety" OR MM "Mathematics Anxiety" OR MM "Performance Anxiety" OR MM "Social Anxiety" OR MM "Speech Anxiety" OR MM "Test Anxiety" OR MM "Anxiety Disorders" OR MM "Castration Anxiety" OR MM "Generalized Anxiety Disorder" OR MM "Obsessive Compulsive Disorder" OR MM "Panic Attack" OR MM "Panic Disorder" OR MM "Phobias" OR MM "Separation Anxiety Disorder" OR MM "Trichotillomania" OR MM "Generalized Anxiety Disorder" OR MM "Speech Anxiety" OR MM "Castration Anxiety" OR MM "Anxiety Sensitivity" OR MM "Test Anxiety" OR MM "Social Anxiety" OR MM "Performance Anxiety" OR MM "Mathematics Anxiety" OR MM "Health Anxiety" OR MM "Death Anxiety" OR MM "Computer Anxiety" View Results (93,763)

S4 MM "Major Depression" OR MM "Anaclitic Depression" OR MM "Dysthymic Disorder" OR MM "Endogenous Depression" OR MM "Late Life Depression" OR MM "Postpartum Depression" OR MM "Reactive Depression" OR MM "Recurrent Depression" OR MM "Treatment Resistant Depression" OR MM "Postpartum Depression" OR MM "Treatment Resistant Depression" OR MM "Late Life Depression" OR MM "Recurrent Depression" OR MM "Reactive Depression" OR MM "Endogenous Depression" OR MM "Anaclitic Depression" OR MM "Dysthymic Disorder" View Results (112,337)

S3 MM "Dementia" OR MM "AIDS Dementia Complex" OR MM "Dementia with Lewy Bodies" OR MM "Presenile Dementia" OR MM "Pseudodementia" OR MM "Semantic Dementia" OR MM "Senile Dementia" OR MM "Vascular Dementia" OR MM "Vascular Dementia" OR MM "Presenile Dementia" OR MM "Alzheimer's Disease" OR MM "Creutzfeldt Jakob Syndrome" OR MM "Picks Disease" OR MM "Senile Dementia" OR MM "Senile Psychosis" OR MM "Semantic Dementia" OR MM "Dementia with Lewy Bodies" OR MM "AIDS Dementia Complex" OR MM "Pseudodementia ... View Results (72,058)

S2 alzheimer View Results (71,569)

S1 MM "Alzheimer's Disease" View Results (42,891)

**CINAHL**

S12 S9 AND S10 Narrow by Language: - english View Results (4,292)View DetailsEdit

S11 S9 AND S10 View Results (4,420)

S10 S4 OR S5 OR S6 OR S7 OR S8 View Results (234,561)

S9 S1 OR S2 OR S3 View Results (61,433)

S8 (MM "Stress, Psychological+") View Results (53,500)

S7 "depressive disorder" View Results (71,905)

S6 "anx*" View Results (112,661)

S5 (MM "Anxiety+") OR (MM "Anxiety Disorders+") View Results (53,514)

S4 (MM "Depression+") View Results (73,260)

S3 (MM "Dementia+") View Results (59,357)

S2 "alzheimer" View Results (28,553)

S1 (MM "Alzheimer's Disease") View Results (25,627)
