# Supplementary material for: Anxiety and depression in Alzheimer’s disease: a systematic review of pathogenetic mechanisms and relation to cognitive decline
Source: Neurol Sci. 2022 Apr 23;43(7):4107–24. doi: 10.1007/s10072-022-06068-x (PMC9213384; doi:10.1007/s10072-022-06068-x)
Supplement: Supplementary file 2 — Supplementary file2 (DOCX 43 KB) [file 10072_2022_6068_MOESM2_ESM.docx]

**Title:** Anxiety and depression in Alzheimer’s disease: a systematic review of pathogenetic mechanisms and relation to cognitive decline.

**Journal name:** Neurological Sciences

**Authors and affiliations:**

**Rossana Botto^1,2^, Nicoletta Callai^2^, Aurora Cermelli^3^, Lorenzo Causarano^4^, Innocenzo Rainero^3^**

^1^Department of Neuroscience, University of Turin, Torino, Italy

^2^Clinical Psychology Unit, “Città della Salute e della Scienza di Torino” Hospital of Turin, Torino, Italy

^3^Aging Brain and Memory Clinic, Department of Neuroscience, University of Turin, Torino, Italy

^4^Biblioteca Federata di Medicina “Ferdinando Rossi”, University of Turin, Torino, Italy

**Corresponding author’s e-mail:**

rossana.botto@unito.it

Figure 1. Flow diagram about the selection procedure.

**Identification of studies via databases and registers**

Duplicate records removed after removing duplicates (n = 3888)

Records identified from:

Databases (Total=14760):

PsycINFO (n=4700);

Embase (n=1351);

Ovid (n=4738);

CINAHL (n=3971)

**Identification**

Records excluded

(n = 10798)

Records screened

(n = 10872)

**Screening**

Reports excluded after full text screening: 40

Reports assessed for eligibility

(n = 74)

Studies included in review

(n = 34)

**Included**
