# Supplementary material for: Anxiety and depression in Alzheimer’s disease: a systematic review of pathogenetic mechanisms and relation to cognitive decline
Source: Neurol Sci. 2022 Apr 23;43(7):4107–24. doi: 10.1007/s10072-022-06068-x (PMC9213384; doi:10.1007/s10072-022-06068-x)
Supplement: Supplementary file 3 — Supplementary file3 (DOCX 42 KB) [file 10072_2022_6068_MOESM3_ESM.docx]

**Title:** Anxiety and depression in Alzheimer’s disease: a systematic review of pathogenetic mechanisms and relation to cognitive decline.

**Journal name:** Neurological Sciences

**Authors and affiliations:**

**Rossana Botto^1,2^, Nicoletta Callai^2^, Aurora Cermelli^3^, Lorenzo Causarano^4^, Innocenzo Rainero^3^**

^1^Department of Neuroscience, University of Turin, Torino, Italy

^2^Clinical Psychology Unit, “Città della Salute e della Scienza di Torino” Hospital of Turin, Torino, Italy

^3^Aging Brain and Memory Clinic, Department of Neuroscience, University of Turin, Torino, Italy

^4^Biblioteca Federata di Medicina “Ferdinando Rossi”, University of Turin, Torino, Italy

**Corresponding author’s e-mail:**

rossana.botto@unito.it

**Newcastle – Ottawa Quality Assesment Scale**

**Etiopathogenetic hypothesis on anxiety and depression in Alzheimer’s disease: a systematic review**

| Author | Selection | Comparability | Exposure/ Outcome | Total |
| --- | --- | --- | --- | --- |
| Amieva (2008) | 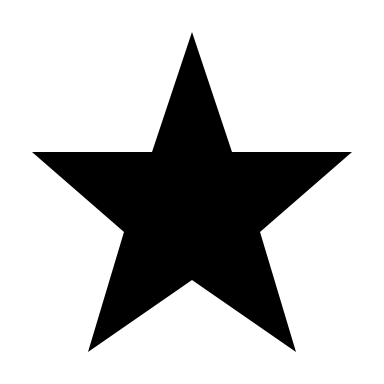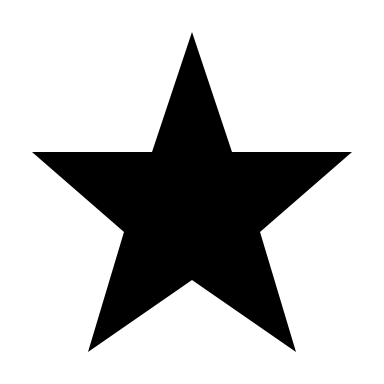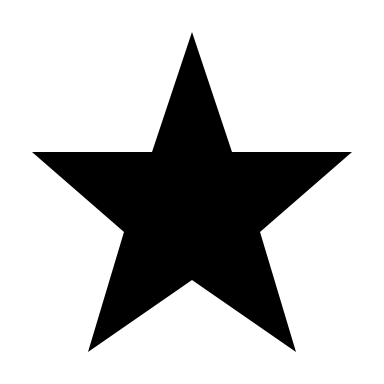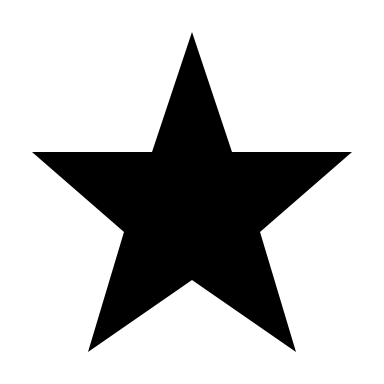 | 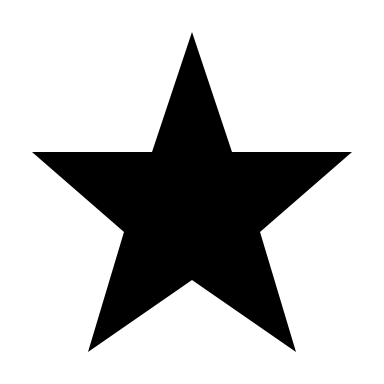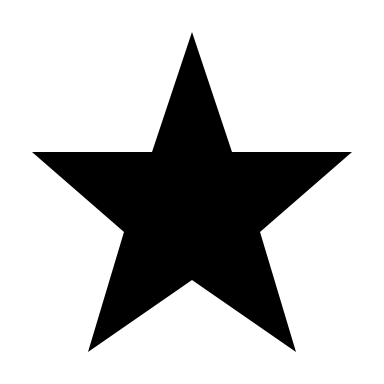 | 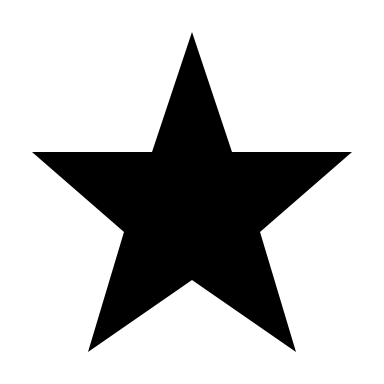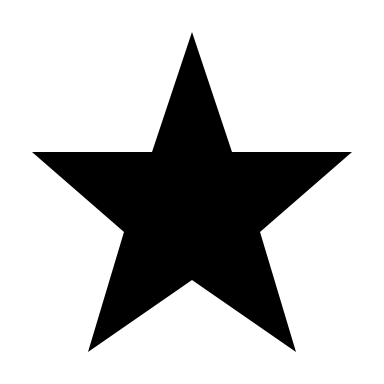 | 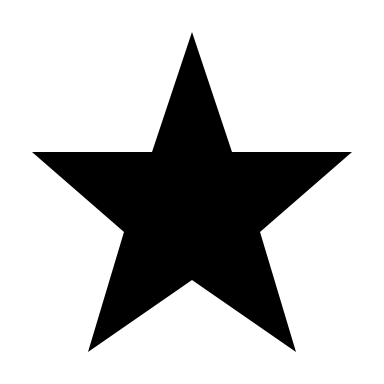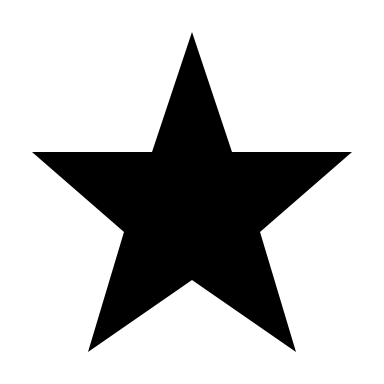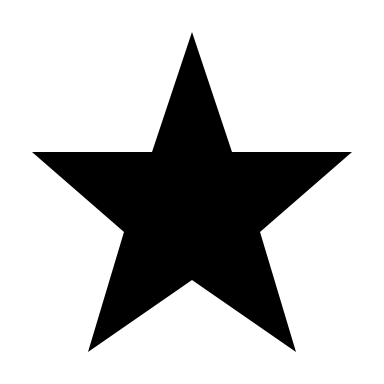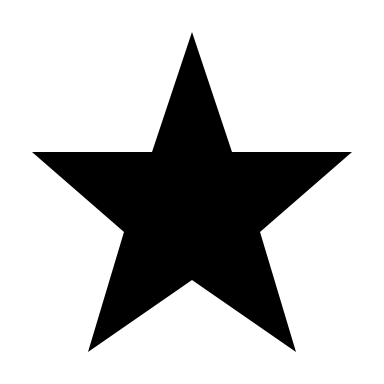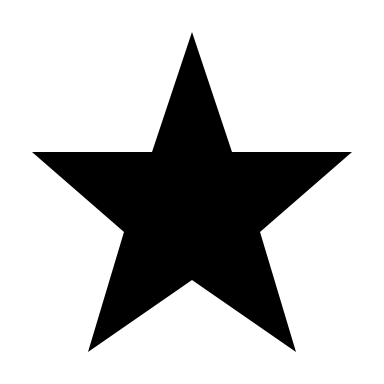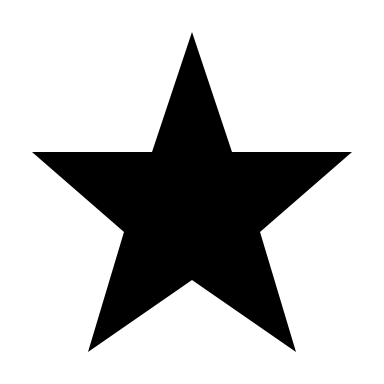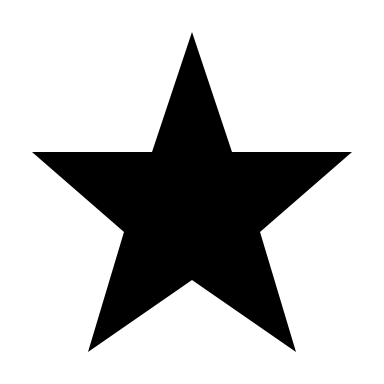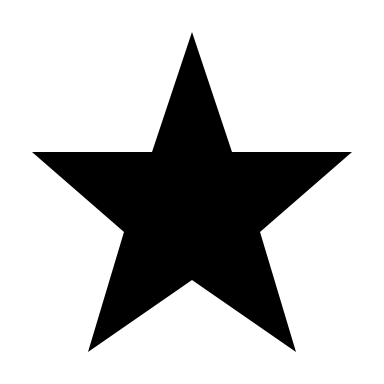 |
| Baillon (2019) | 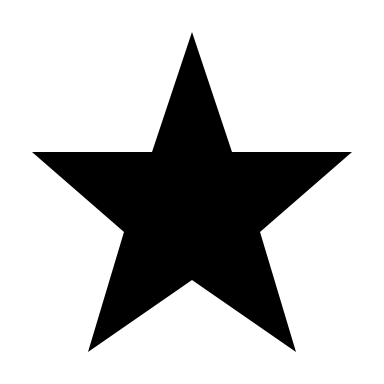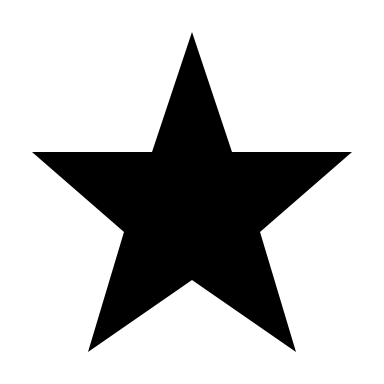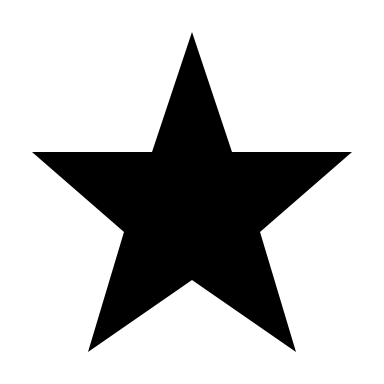 | 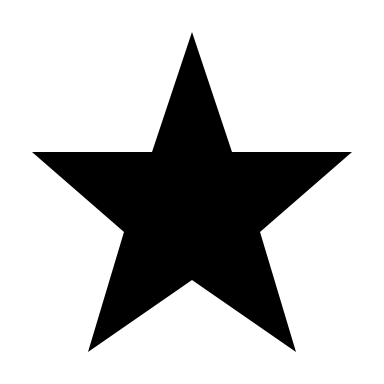 | 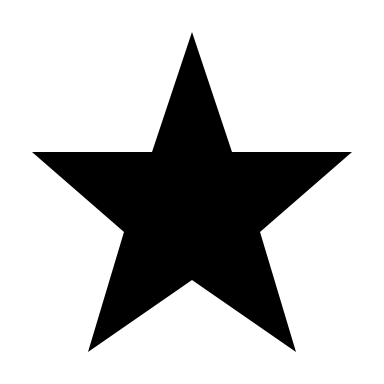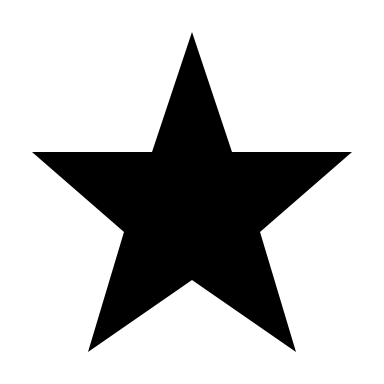 | 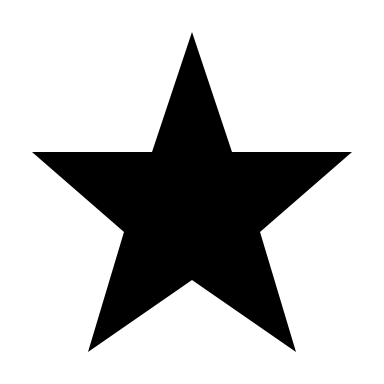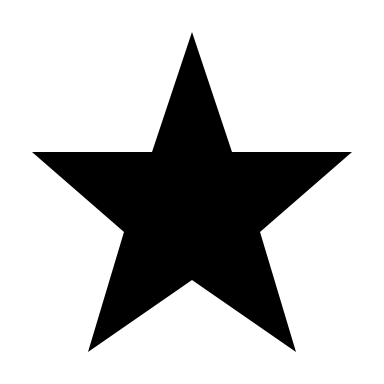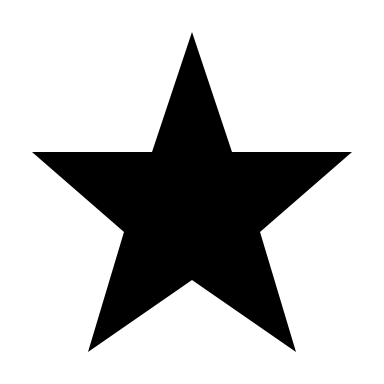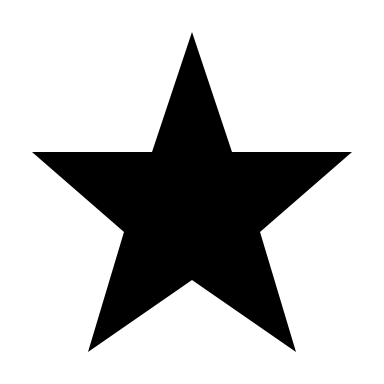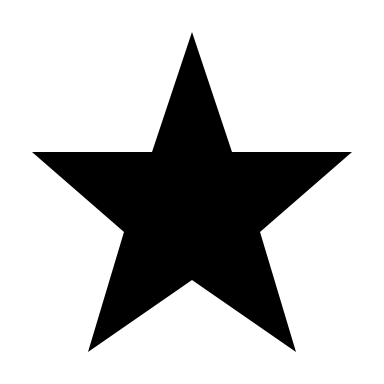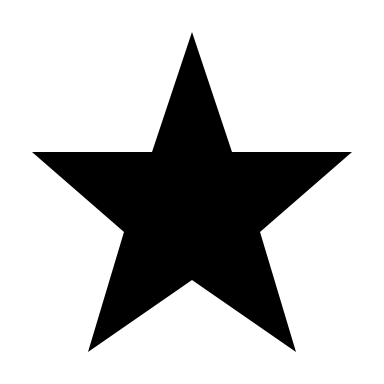 |
| Banning (2020) | 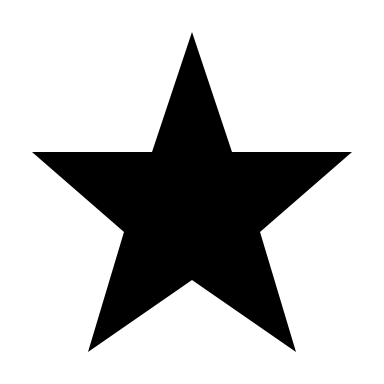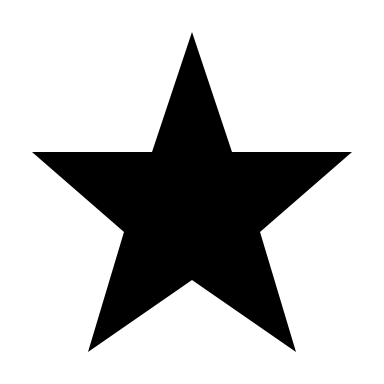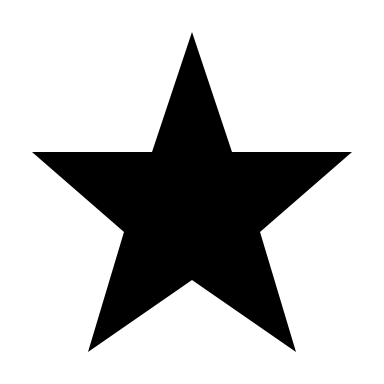 | 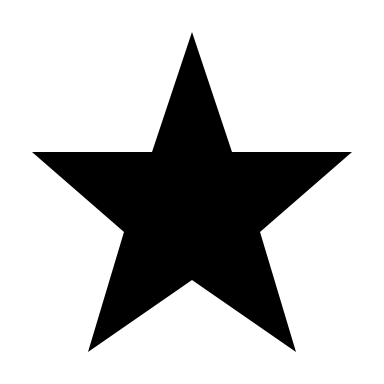 | 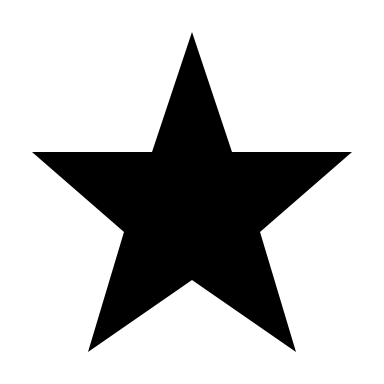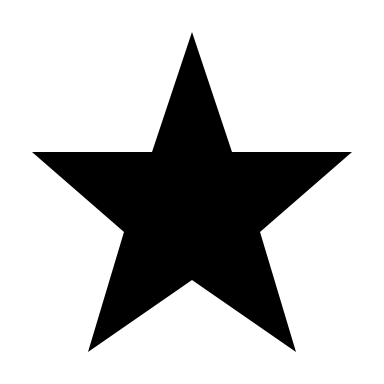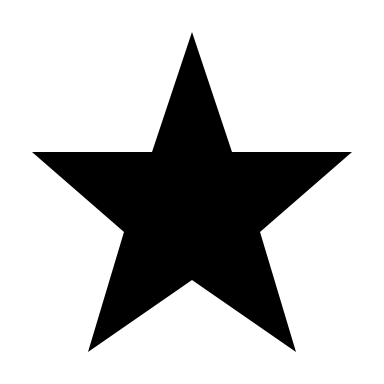 | 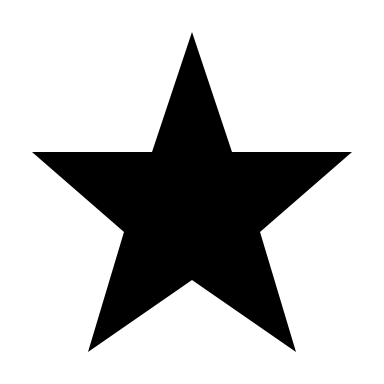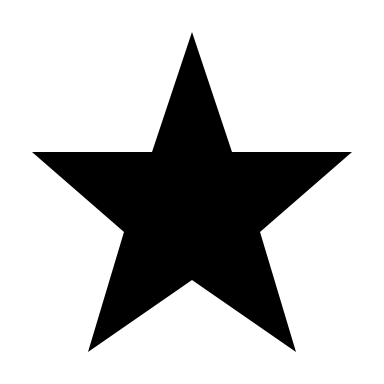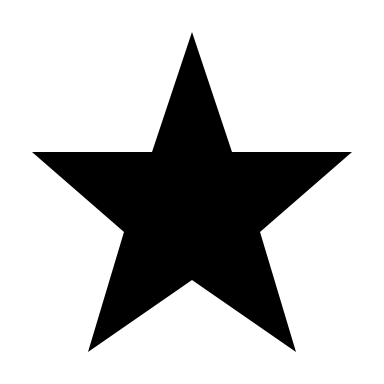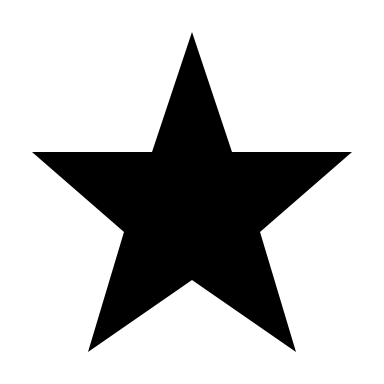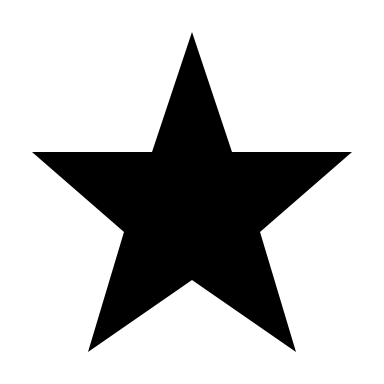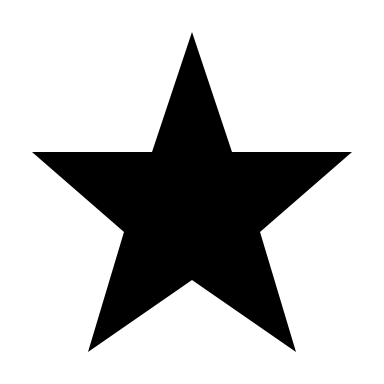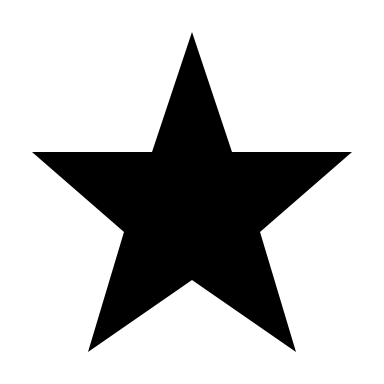 |
| Banning (2021) | 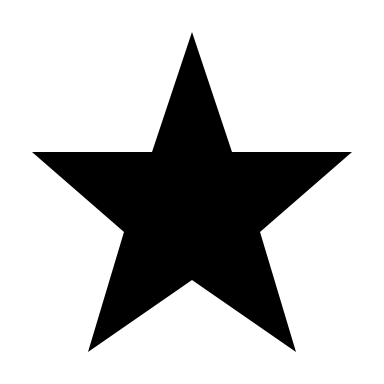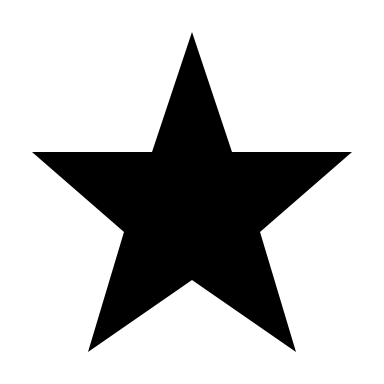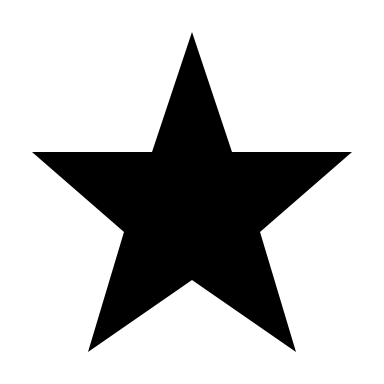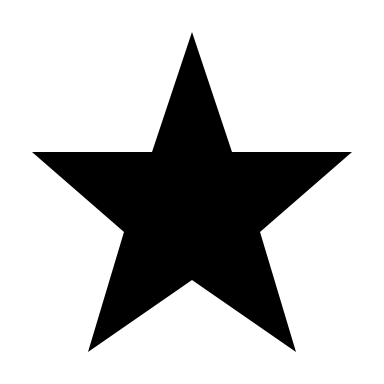 | 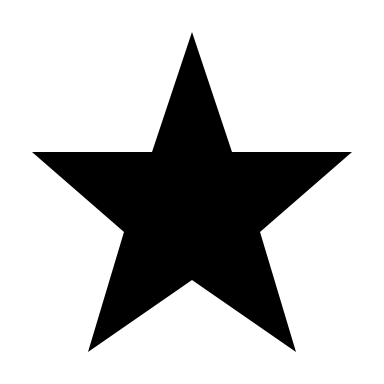 | 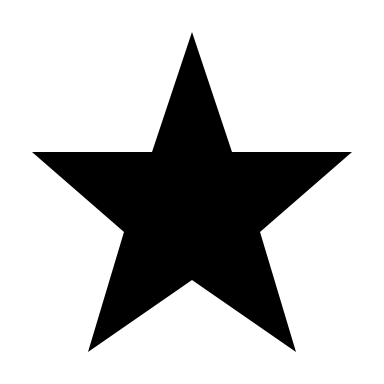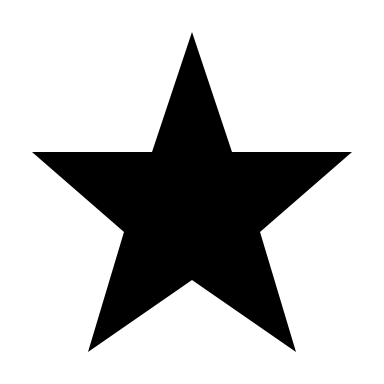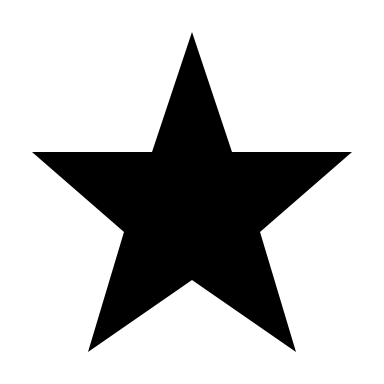 | 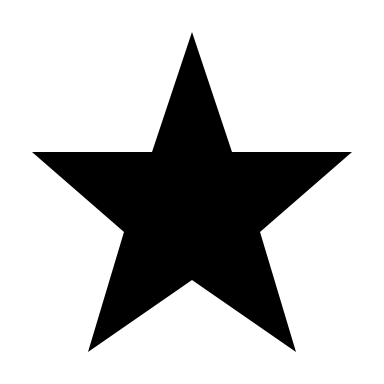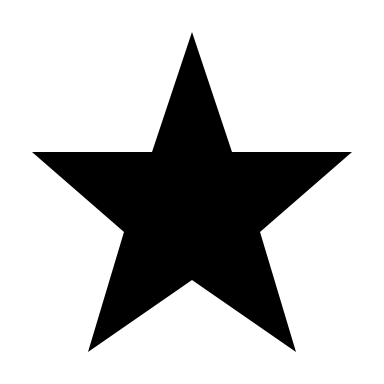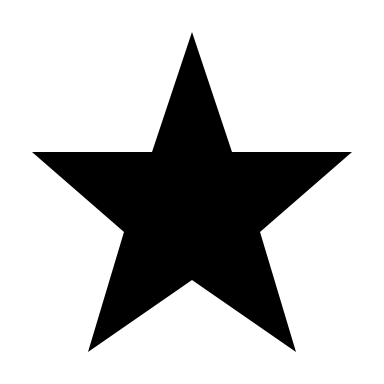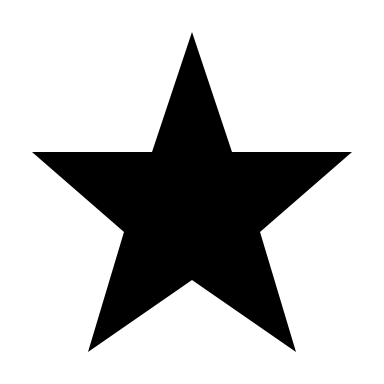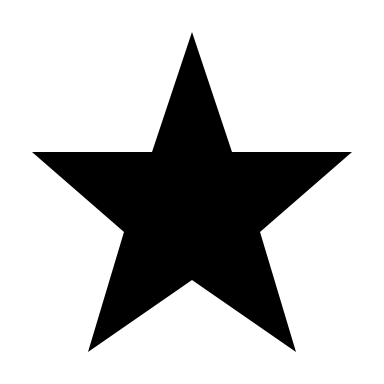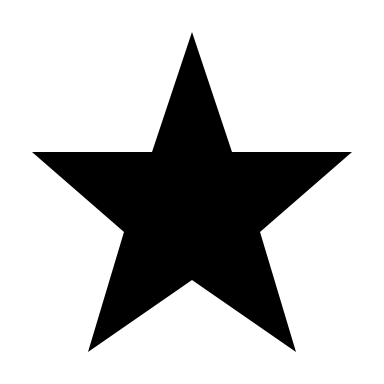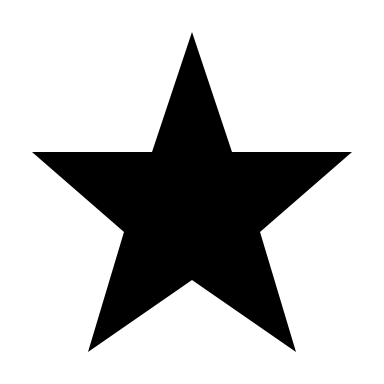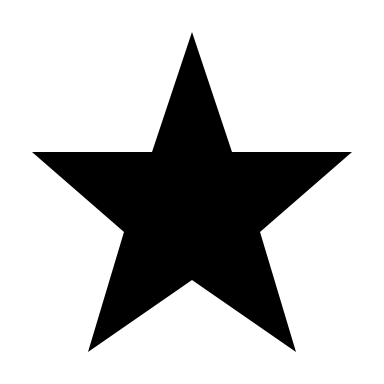 |
| Barca (2017) | 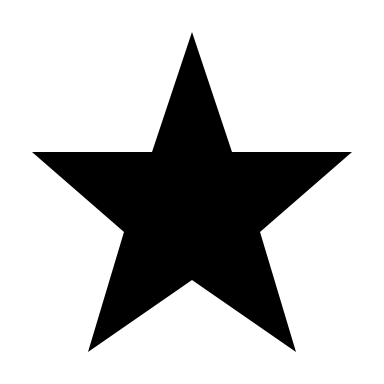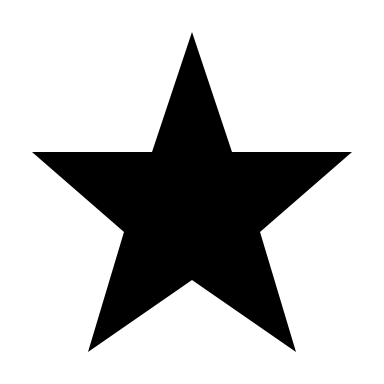 | 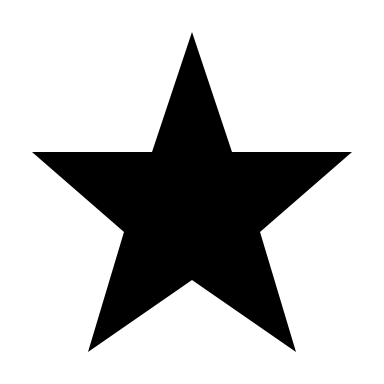 | 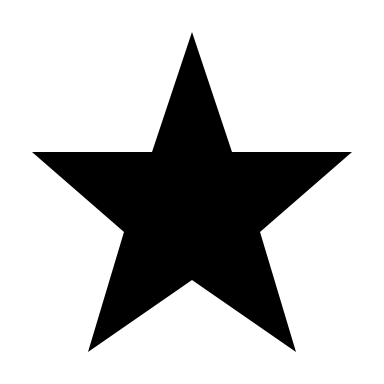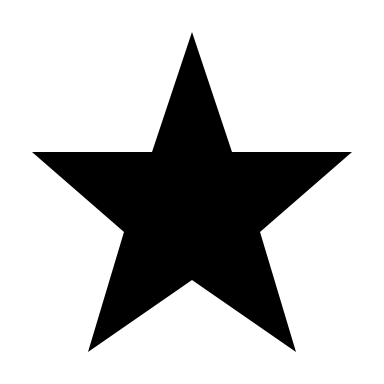 | 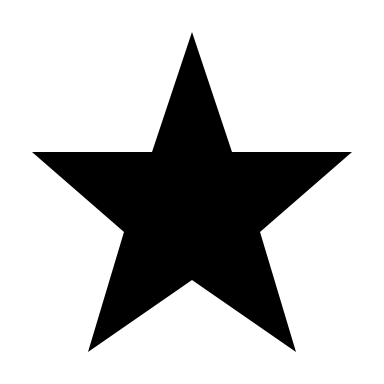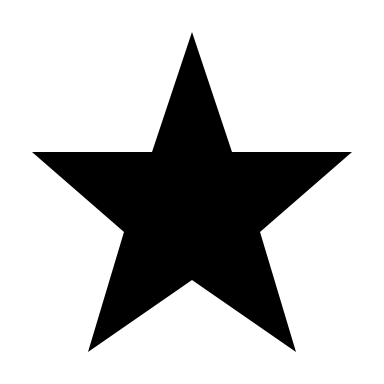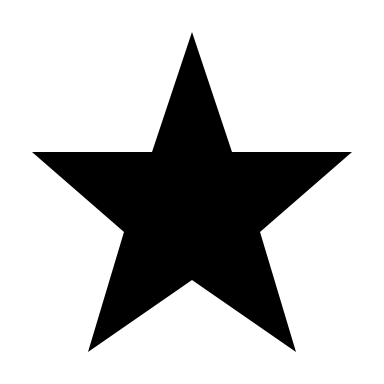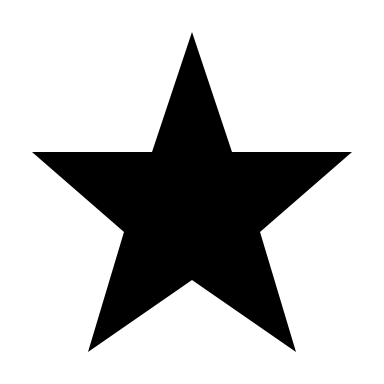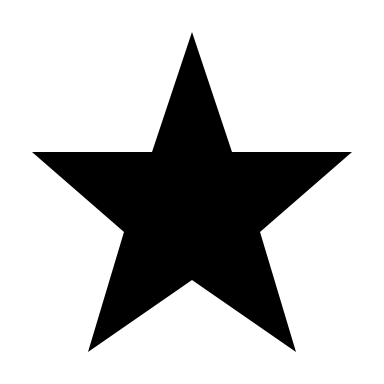 |
| Cannon-Spoor (2005) | 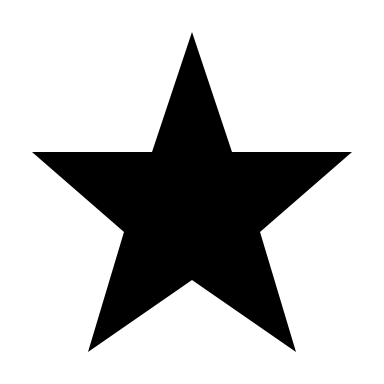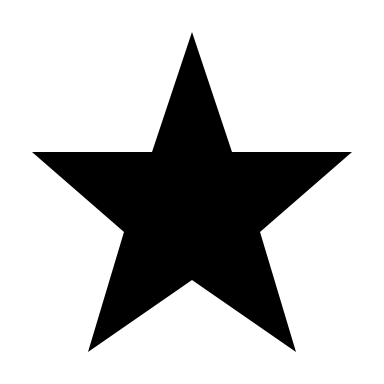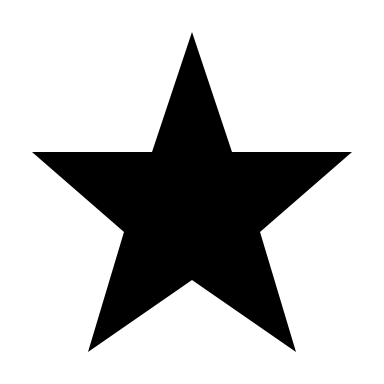 | 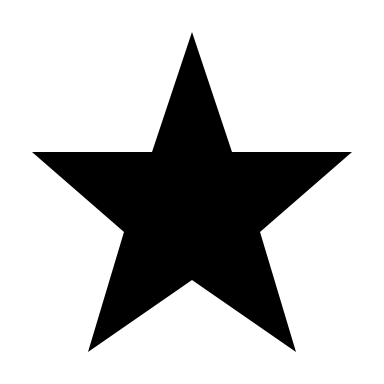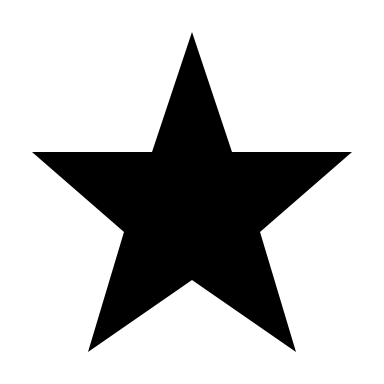 | 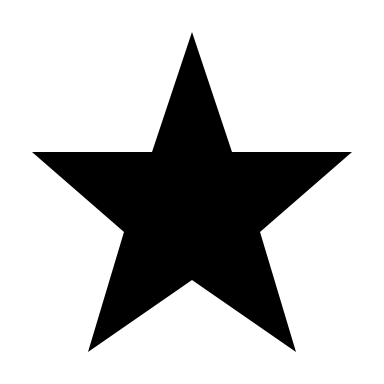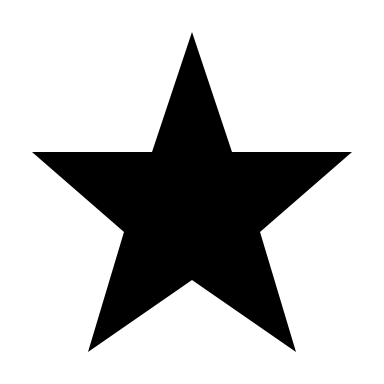 | 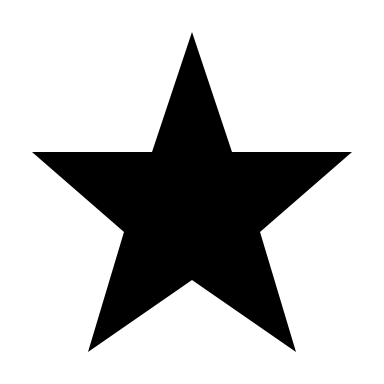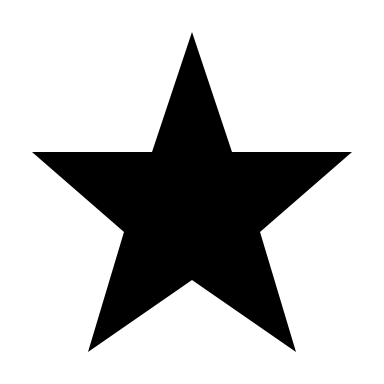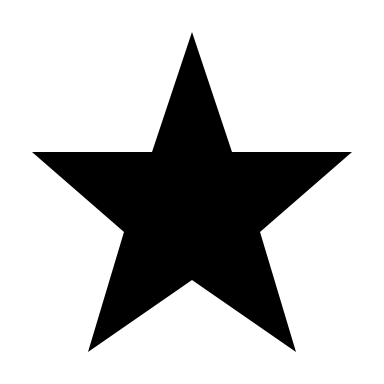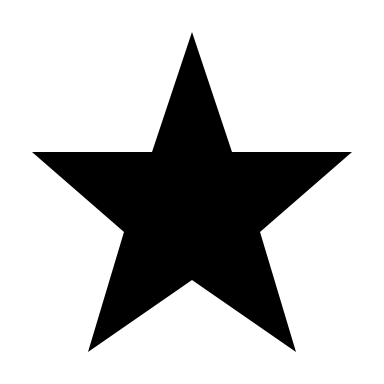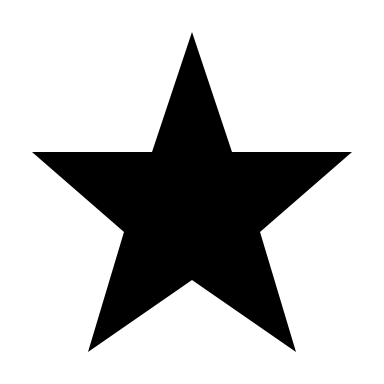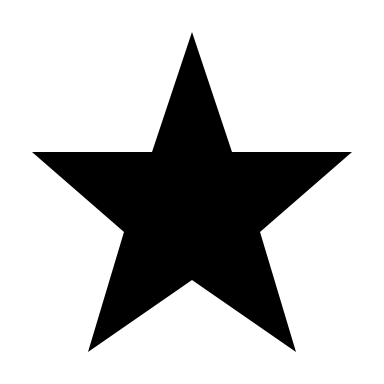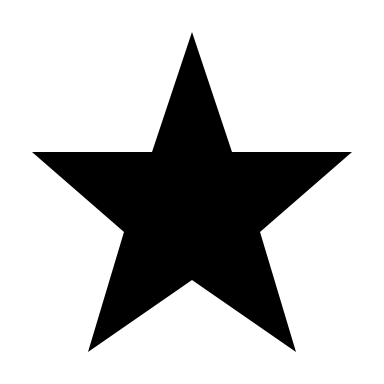 |
| Chemerinski (1998) | 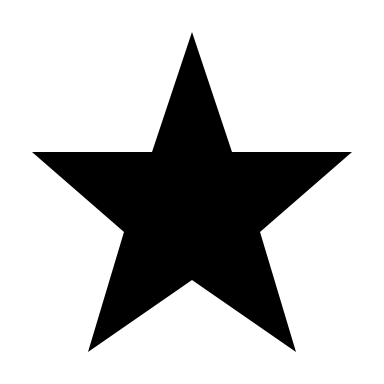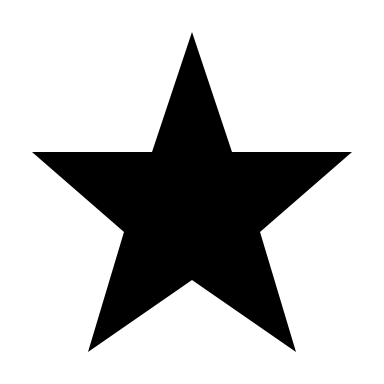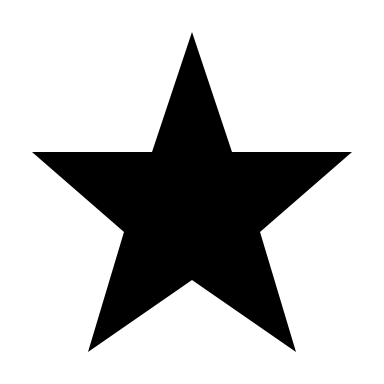 | 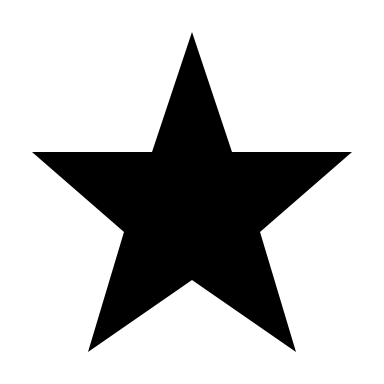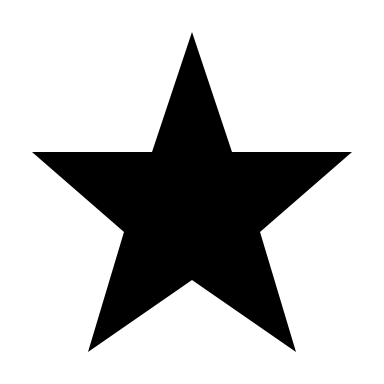 | 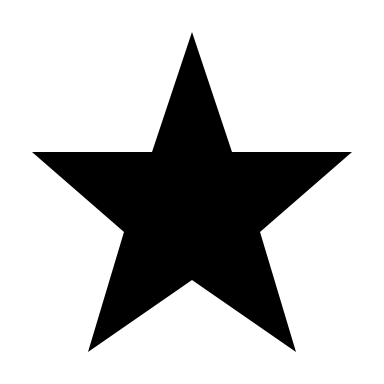 | 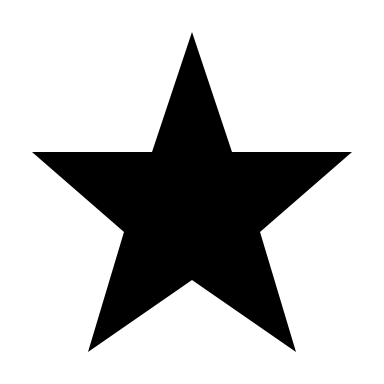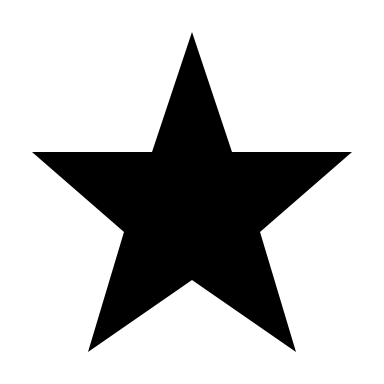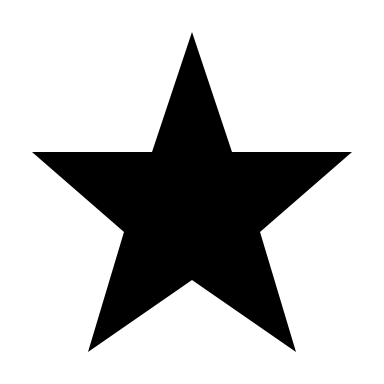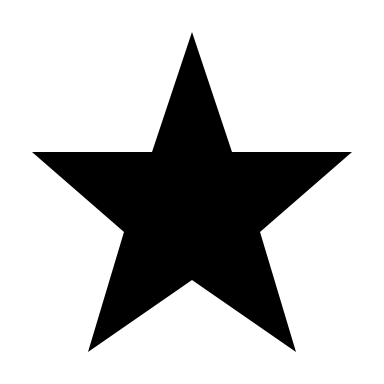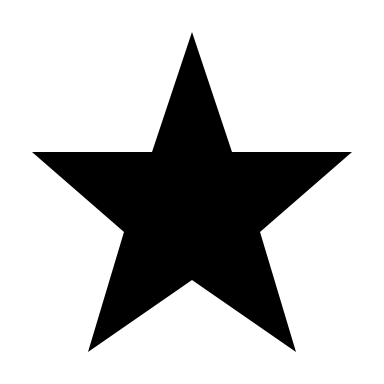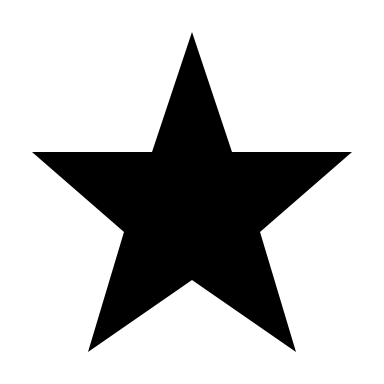 |
| Förstl (1992) | 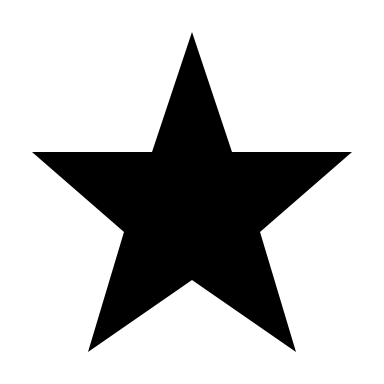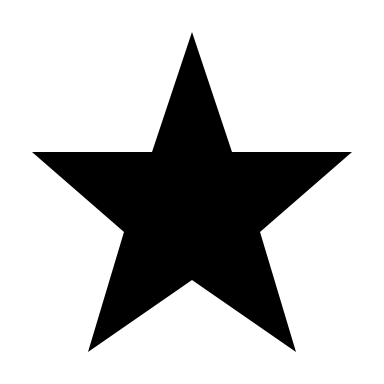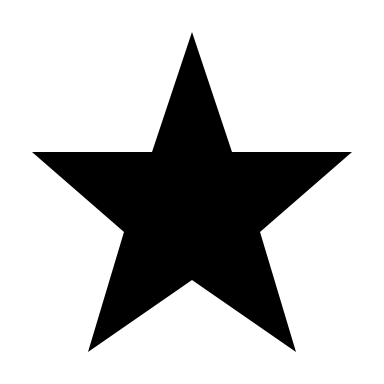 | 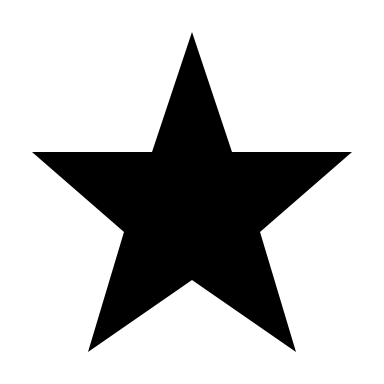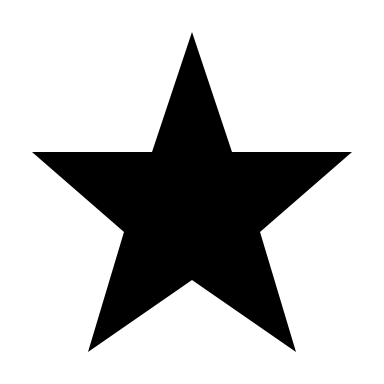 | 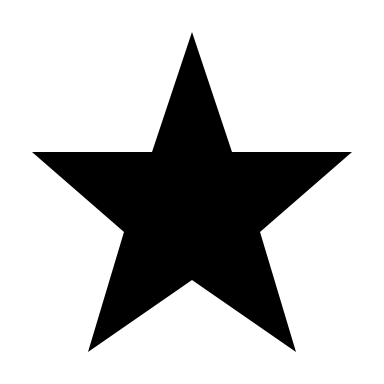 |  |
| Gilley (2004) |  |  |  |  |
| Hashimoto (2006) |  |  |  |  |
| Haupt (1995) |  |  |  |  |
| Heun (2002) |  |  |  |  |
| Holtzer (2005) |  |  |  |  |
| Kaiser (2014) |  |  |  |  |
| Lebedeva (2014) |  |  |  |  |
| Lopez (1990) |  |  |  |  |
| Meynen (2009) |  |  |  |  |
| Migliorelli (1995) |  |  |  |  |
| Milwain (2005) |  |  |  |  |
| Panegyres (2014) |  |  |  |  |
| Pearlson (1990) |  |  |  |  |
| Rouch (2019) |  |  |  |  |
| Spalletta (2012) |  |  |  |  |
| Tagai (2014) |  |  |  |  |
| Tanaka (2015) |  |  |  |  |
| Tsang (2008) |  |  |  |  |
| Van Vliet (2012) |  |  |  |  |
| Wilson (2003) |  |  |  |  |
| Wilson (2008) |  |  |  |  |
| Wilson (2010) |  |  |  |  |
| Wu (2020) |  |  |  |  |
| Zahodne (2013) |  |  |  |  |
| Zubenko (2003) |  |  |  |  |
| Zweig (1988) |  |  |  |  |
